# Enhanced deep Convolutional Neural Network for SARS-CoV-2 variants classification

**DOI:** 10.3389/frai.2025.1512003

**Published:** 2025-09-08

**Authors:** Olaitan I. Awe, Hesborn Obura, Charles Ssemuyiga, Evans Mudibo, Mike J. Mwanga

**Affiliations:** ^1^African Society for Bioinformatics and Computational Biology, Cape Town, South Africa; ^2^Department of Computer Science, Faculty of Science, University of Ibadan, Ibadan, Nigeria; ^3^Department of Biochemistry and Biotechnology, School of Pure and Applied Science, Pwani University, Kilifi, Kenya; ^4^Pwani University Biosciences Research Centre, Pwani University, Kilifi, Kenya; ^5^PharmaQsar Bioinformatics Firm, Kampala, Uganda; ^6^Department of Biological and Environmental Sciences, School of Natural Sciences, Kampala International University, Kampala, Uganda; ^7^Centre for Geographic Medicine Research, Kenya Medical Research Institute Wellcome Trust Research Program, Kilifi, Kenya

**Keywords:** SARS-CoV-2, machine learning, genomics, deep learning, Convolutional Neural Networks, spike gene

## Abstract

**Introduction:**

Rapid and scalable classification of SARS-CoV-2 genomes from spike-gene sequences can support real-time genomic surveillance in contexts where whole-genome data or high-end computing resources are limited.

**Methods:**

We curated approximately 35,800 quality-filtered spike sequences spanning multiple clades and lineages and trained a hybrid CNN–BiLSTM model with standard regularization and class-imbalance handling. Model performance was benchmarked against Nextclade assignments and compared with classical machine-learning baselines.

**Results:**

Across 10 experimental runs, the model achieved a mean training accuracy of 99.74% ± 0.11, a validation accuracy of 99.00% ± 0.00, and a test accuracy of 99.91% ± 0.03. In benchmarking against the molecular epidemiology tool Nextclade, our model demonstrated superior performance, correctly identifying 100% of Omicron sequences, compared to 34.95% achieved by Nextclade. Saliency and feature attribution analyses highlighted recurrent spike substitutions consistent with known variant-defining mutations, as well as additional uncharacterized motifs with potential biological relevance.

**Discussion:**

These findings demonstrate that spike-only deep models can provide rapid and accurate clade or variant classification, while also yielding interpretable feature importance. Such models complement phylogenetic approaches in settings with constrained resources and enable efficient triage of samples for confirmatory whole-genome analysis, supporting more timely genomic surveillance.

## 1 Introduction

Taxonomic classification of pathogens is central to clinical diagnosis and outbreak surveillance. Traditional approaches such as PCR and metagenomic sequencing enable pathogen detection without prior knowledge (Li et al., 2021; Omar et al., 2024), and have become increasingly accessible due to reduced costs and improved tools. However, these methods remain computationally demanding and time-consuming, particularly for large-scale datasets, owing to incomplete reference databases and the growing microbial diversity (Menzel et al., 2016). This can delay timely identification during outbreaks and requires substantial bioinformatics expertise. Machine learning-based tools can identify and extract important sequence features for sequence classification in a computationally efficient manner. In a DNA sequence, these features will be the pattern of arrangement of nucleotides in a sequence that is/are unique for each pathogen. For this reason, machine learning methods have been applied in the identification and classification of pathogens in clinical samples (Abd-Alhalem et al., 2021; Liang et al., 2020; Hanson et al., 2024) as well as in complex diseases (Enejoh et al., 2025). For instance, VirFinder (Ren et al., 2017) models used *k-mer* frequency to classify viruses while VirSorter (Roux et al., 2015) uses a probabilistic model tool to predict viral sequences. Randhawa et al. (2020) proposed a supervised machine learning with a digital signal process model for genomic identification of COVID-19 virus signatures, important in the classification of SARS-CoV-2 (Table 1).

**Table 1:** Shared spike protein mutations among major SARS-CoV-2 VOCs, including genomic coordinates (based on NC_045512.2), spike gene nucleotide positions, and their known phenotypic effects.

Mutation	Genomic position	Spike nucleotide position	Variants sharing the mutation	Functional role
D614G	23,403	1841	All VOCs (Alpha, Beta, Gamma, Delta, Omicron)	Enhances spike stability and infectivity (Korber et al., 2020).
N501Y	23,063	1,501	Alpha, Beta, Gamma, Omicron	Increases ACE2 binding affinity
E484K	23,012	1,450	Beta, Gamma (not Delta)	Immune escape from neutralizing antibodies (Greaney et al., 2021).
P681H/R	23,604	2042	Alpha (H), Delta (R), Omicron (H)	Modulates furin cleavage, and spike activation (Saito et al., 2022).
K417N/T	22,813	1,251	Beta (N), Gamma (T), Omicron (N)	Immune evasion via RBD alterations (Greaney et al., 2021).
Δ69–70 deletion	21,765–21,770	203–208	Alpha, Omicron	Disrupts the N-terminal domain, affects antigenicity (McCarthy et al., 2021).

Deep learning (DL), a subfield of machine learning, has emerged as a powerful framework for automated feature extraction from biological sequences, particularly through architectures originally developed for natural language processing. By leveraging multiple non-linear transformations, DL models can learn hierarchical representations of nucleotide or amino acid sequences without the need for handcrafted features (Abd-Alhalem et al., 2021; Gunasekaran et al., 2021). These capabilities have been widely applied in genomics for tasks such as DNA classification, gene annotation, protein structure prediction, and viral detection. Notably, several DL-based tools such as VirHunter (Sukhorukov et al., 2022), DeepVirFinder (Ren et al., 2017), and ViralMiner (Tampuu et al., 2019) have demonstrated high accuracy in identifying viral sequences directly from raw metagenomic data. RNA-seq datasets provide an avenue for the detection of the expression of genes in a sample (Alaya et al., 2024; Ben Aribi et al., 2024, 2025; El Abed et al., 2023; Ather et al., 2018; Die et al., 2019). PACIFIC, another DL framework, was developed to detect co-infections of respiratory viruses including SARS-CoV-2 from RNA-seq datasets (Charles et al., 2023; Elbasir et al., 2023). These models offer substantial advantages over alignment-dependent methods, particularly in their scalability and ability to detect novel or divergent viral genomes (Table 2).

**Table 2:** A summary of the studies compared to the present study.

Study	Acc.	Recall	F1 (Weig.)	F1 (Macro)	Prec.	Reference
Spike2Vec	0.68	0.68	0.64	0.49	0.79	Ali and Patterson (2021)
Kernel Approximation	0.998	0.997	0.998	0.998	0.997	Ali et al. (2021)
PWM2Vec	0.84	0.84	0.85	0.80	0.84	Ali et al. (2022)
Neural Network	0.77	0.77	0.74	0.49	0.78	Ali et al. (2023)
This work (CNN + BiLSTM)	0.99 ± 0.00	0.99 ± 0.00	0.99 ± 0.00	0.99 ± 0.00	0.99 ± 0.00	This work

Only the best values for all used ML algorithms/Classifiers were compared. The results for PWM2Vec reported in this table involved ridge regression as the feature selection approach.

SARS-CoV-2 VOCs are primarily distinguished by recurrent mutations in the spike (S) gene, which encodes the viral protein responsible for mediating host cell entry via the Angiotensin-Converting Enzyme 2 (ACE2) receptor (Nyamari et al., 2023; Nzungize et al., 2022). These mutations not only influence viral transmissibility and immune escape but also serve as robust genomic signatures for lineage classification (Awe et al., 2023; Harvey et al., 2021; Korber et al., 2020; Obura et al., 2022; Oluwagbemi and Awe, 2018). For instance, key substitutions in the receptor-binding domain (RBD) such as N501Y, E484K, and K417N/T alter ACE2 binding affinity and antibody recognition, thereby shaping the phenotypic profiles of Alpha, Beta, Gamma, and Omicron lineages (Cao et al., 2021; Harvey et al., 2021). Meanwhile, mutations such as L452R and P681R in Delta contribute to enhanced infectivity and proteolytic activation (Meng et al., 2022). Although some of these markers are unique to specific variants, several have emerged independently across lineages through convergent evolution, complicating classification tasks (Table 1) (Edara et al., 2021). Notably, D614G became globally dominant early in the pandemic and is now present across all major VOCs, reducing its discriminatory power (Korber et al., 2020). Similarly, the N501Y substitution, once a hallmark of Alpha, also appears in Beta, Gamma, and Omicron (Harvey et al., 2021). These shared features can blur the mutational boundaries between variants, particularly in models relying solely on sequence-based inputs. Deep learning models, however, can implicitly learn to differentiate subtle mutation contexts and adjacent sequence motifs. Several studies have demonstrated that the spike gene alone is sufficient for robust lineage classification due to its high mutational density and selective pressure (Harvey et al., 2021). Furthermore, recombination signals that manifest in spike often reflect broader genome-wide signatures, making spike-based models a reliable first-tier diagnostic filter.

### 1.1 Existing characterization methods for SARS-CoV-2 spike sequence classification

Traditional SARS-CoV-2 variant classification approaches typically rely on structured feature encodings such as k-mer frequencies or one-hot encoding (OHE), followed by classical machine learning algorithms which typically require explicit feature engineering and perform well with structured inputs such as k-mer or one-hot encoded sequences, they include; Support Vector Machines (SVM), Decision Trees (DT), Random Forests (RF), Logistic Regression (LR), k-Nearest Neighbors (KNN), and Naive Bayes (NB) (Kuzmin et al., 2020; Nicora et al., 2022). The k-mer encoding technique transforms biological sequences into fixed-length numerical vectors by segmenting nucleotide or amino acid strings into overlapping sub-sequences of length *k*. This alignment-free approach captures local motif frequency information and has been widely applied in genomic classification tasks, particularly when sequence alignment is computationally infeasible. For example, Kuzmin et al. (2020) applied Principal Component Analysis (PCA) and OHE to spike sequences, achieving >98% accuracy using SVM, LR, and DT models. Nicora et al. (2022) employed amino acid k-mers in a one-class classification system that detected emerging variants with >98% precision. Ali et al. (2021) introduced a kernel approximation technique for k-mer-based feature vectors, showing improved accuracy even on limited training data. Other notable methods include Spike2Vec, which computes amino acid embeddings using information gain to highlight biologically informative residues (Ali and Patterson, 2021), and PWM2Vec, a position-weight matrix-based embedding for capturing positional nucleotide importance (Ali et al., 2022). These approaches support classical classifiers but may suffer performance loss on complex, full-length genomic inputs. Further innovations have combined sequence encoding with neural network architectures. Ali et al. (2023) presented a simple neural network using k-mers as input, demonstrating strong classification by geographic lineage and identifying informative residues. Nan et al. (2022) proposed a fine-grained adaptation of a sequence-based DL model that showed clear inter-lineage and intra-lineage clustering in Omicron sequences. Similarly, Li et al. (2022) developed a 3D Convolutional Neural Network (CNN) that used dinucleotide composition to differentiate Beta, Delta, and Omicron lineages with high predictive power (Table 2).

OHE leads to high-dimensional sparse vectors, which strain computational resources and are prone to overfitting, while k-mer-based encodings neglect the positional order and long-distance dependencies between biologically significant motifs. These limitations are further amplified in the context of recombinant or drifted variants, where subtle shifts in mutation patterns require models capable of deeper contextual understanding. To overcome these challenges, we designed a hybrid deep learning architecture that combines CNNs with BiLSTMs, trained end-to-end on aligned, full-length spike DNA sequences. CNNs are deep learning architectures capable of automatically extracting hierarchical spatial features from structured data, they have proven effective for detecting spatially localized sequence features such as conserved motifs or mutation clusters through trainable filters that enable hierarchical pattern learning without manual feature engineering (Gunasekaran et al., 2021; Zeng et al., 2016). This makes them well-suited for extracting discriminative subsequence patterns embedded within high-throughput genomic data. Following the convolutional layers, we integrated BiLSTMs to capture long-range dependencies by processing the input sequence in both forward and backward directions. Unlike conventional Recurrent neural networks (RNNs), BiLSTMs, a class of RNNs utilize gated units that control the flow of information across time steps, preserving critical contextual relationships among nucleotide positions (Graves, 2012; Hochreiter and Schmidhuber, 1997). This is especially relevant in genomics, where the impact of mutations often depends on their broader sequence environment. By aligning and encoding complete spike sequences, our model can learn variant-specific signatures in both conserved and variable regions. In doing so, it mitigates common pitfalls of traditional ML models, offering a scalable, accurate, and biologically informed solution for genomic variant classification.

## 2 Materials and methods

### 2.1 Code availability

All code, model training scripts, and preprocessing pipelines used in this study are publicly available in the GitHub repository (https://github.com/omicscodeathon/ml_sarscov2) to ensure full transparency and reproducibility of the results.

### 2.2 Python libraries and computational tools

**Figure 1:**
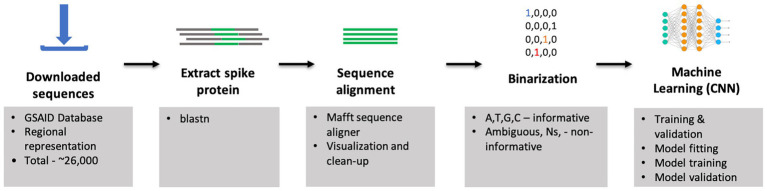
Data preprocessing workflow: feature extraction, feature encoding, and classifications. The first step is to input raw sequences for feature extraction and feature encoding using the one-hot encoding technique. Then, the one-hot encoded sequences are fed to the CNN model for classification analysis.

All analyses and model development were implemented in Python (version: 3.12.7), leveraging a range of established open-source libraries optimized for deep learning, scientific computing, and bioinformatics workflows. Model training and evaluation were conducted using TensorFlow (version: 2.19.0) and Keras (version: 3.10.0), which provide a high-level API for defining and training deep neural networks. The CNN–BiLSTM architecture was constructed using the Keras Sequential API, with additional functionality for dropout, batch normalization, and L2 regularization to enhance generalization performance (Gulli and Pal, 2017; Ramasubramanian and Singh, 2019; Sergeev and Del Balso, 2018). For numerical operations and data manipulation, NumPy (version: 1.26.4) and Pandas (version: 2.2.2) were employed. NumPy was used for efficient matrix operations on encoded sequence arrays, while Pandas facilitated structured handling of metadata such as variant labels and FASTA sequence records (Gupta and Bagchi, 2024). Matplotlib (version: 3.9.2) and Seaborn (version: 0.13.2) were used extensively for data visualization, including model training curves, confusion matrices, and distribution plots (Lemenkova, 2020). Evaluation metrics including precision, recall, F1-score, accuracy, and log loss were computed using functions from Scikit-learn (version: 1.6.1), generation of classification reports, and confusion matrices with multi-class support (Kramer, 2016). To streamline file handling, OS was used for automated directory creation and result storage. This modular and transparent Python-based implementation ensures reproducibility, facilitates extension to other genomic datasets, and supports integration into scalable bioinformatics pipelines (Pawar et al., 2024; Coetzer et al., 2025). Model training was conducted on a high-performance Intel Xeon Linux system equipped with 18 cores and 512 GB RAM. TensorFlow Lite was utilized to convert the trained model into a lightweight, quantized format suitable for deployment on resource-constrained devices (Jacob et al., 2018) (Figure 1).

### 2.3 Dataset collection and spike gene extraction

**Figure 2:**
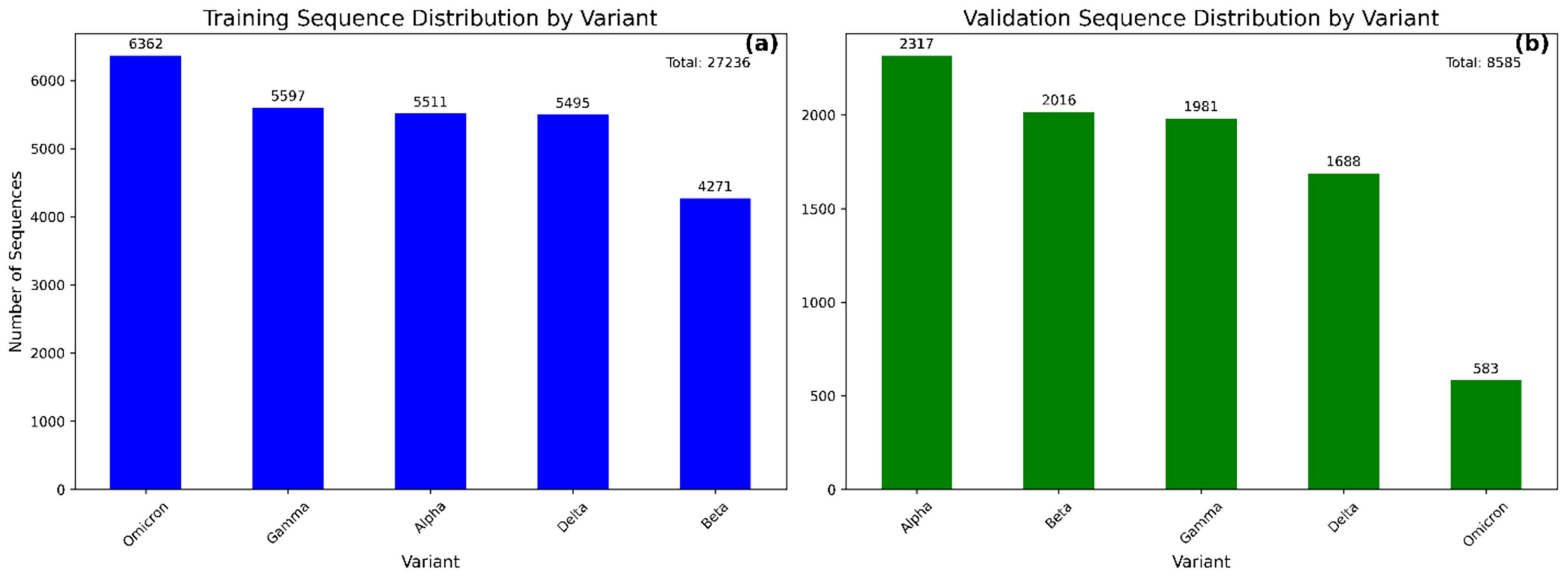
Bar plots showing the distribution of SARS-CoV-2 variant sequences in the modeling **(a)** and validation **(b)** datasets. Each bar represents the number of sequences associated with a specific variant. This distribution highlights the class balance across datasets used for model training and evaluation.

To construct a variant classification model, we downloaded >26,000 representative, high-quality SARS-CoV-2 whole-genome sequences from the GISAID database (https://www.gisaid.org/) using the following inclusion criteria: “complete genome,” “high coverage,” “collection date complete,” and sampling dates between 01/01/2021 and 08/04/2022. Sequences were selected with the representation of pre-defined geographical regions (Africa, Asia, Oceania, S. America, N. America) to ensure global representation and reduce regional sampling bias, thereby enhancing the model’s generalizability across diverse SARS-CoV-2 variant distributions. More diverse validation and omicron training Data of 10,000 sequences was downloaded from NCBI Virus (https://www.ncbi.nlm.nih.gov/labs/virus/vssi/#/). Complete SARS-CoV-2 genome sequences were downloaded and compiled into a local nucleotide BLAST database using the makeblastdb utility from the BLAST+ suite (Camacho et al., 2009). To extract the spike gene from each genome, we used the spike gene sequence from the Wuhan reference genome (NC_045512.2 / Wuhan-Hu-1) as a query in a local BLASTn search. To ensure high specificity, each BLASTn search was restricted to a single high-scoring segment pair (HSP) per subject sequence, and the number of target sequences was set to match the size of the database. This alignment-based extraction strategy enabled high-confidence localization of spike regions across diverse genomic backgrounds. Results were output in tabular format, and genomic coordinates triplets (sequence ID, start, stop) were programmatically extracted using a custom Python script. Only alignments with lengths >3,000 bp were retained to ensure near-complete spike gene coverage, consistent with the expected gene length (~3,800 bp) (Zhou et al., 2020). Spike gene segments were then extracted from the alignment coordinate sets and extracted spike gene sequences were then aligned using MAFFT v7.475 (Katoh et al., 2019). This alignment step standardized sequence length and preserved homologous nucleotide positions across samples (Training and validation), ensuring compatibility with downstream convolutional models. The distribution of training and validation data is shown in Figure 2.

### 2.4 Feature construction and dataset preparation

We performed systematic preprocessing to convert aligned spike gene sequences and labels into a supervised learning format compatible with deep learning. To ensure full reproducibility, the aligned FASTA files were parsed into structured Pandas DataFrames. Headers were preformatted to embed SARS-CoV-2 variant labels (Alpha, Beta, Gamma, Delta, Omicron), which were extracted and mapped to integer class labels from 0 to 4. All nucleotide sequences were capitalized to maintain consistency in encoding.

#### 2.4.1 One-hot encoding of nucleotide sequences

We implemented a bespoke one-hot encoding routine to convert each nucleotide in the spike gene into a binary matrix suitable for input into a Convolutional Neural Network, a widely adopted approach in genomic deep learning (Zeng et al., 2016). The encoding scheme employed was A = [1, 0, 0, 0], C = [0, 1, 0, 0], G = [0, 0, 1, 0], T = [0, 0, 0, 1], and Ambiguous bases (e.g., N, R, Y) and gaps were considered non-informative and assigned [0, 0, 0, 0]. This masking strategy reflects the uncertainty inherent in these positions and avoids assigning fractional values that could introduce noise and reduce model interpretability (Juan et al., 2017). Insertions were treated as valid bases, whereas deletions were treated similarly to ambiguous characters and ignored during encoding (Zeng et al., 2016). The resulting sequences were transformed into a 3D NumPy array with dimensions corresponding to the number of samples, aligned nucleotide positions, and four binary channels representing the DNA bases (A, C, G, T) (Juan et al., 2017). This array served as the input feature matrix for model training. The corresponding variant labels were extracted and mapped to integer values/labels ranging from 0 to 4 (Gamma = 0, Delta = 1, Beta = 2, Alpha = 3, Omicron = 4) which were used as the ground-truth target classes in the supervised classification framework (Figure 1).

### 2.5 Model architecture, training, and evaluation

#### 2.5.1 CNN-based feature extraction

We used four sequential Conv1D layers with 54, 27, 14, and 7 filters, respectively, and kernel sizes progressively reduced from 6 to 2. The input layer receives a 3D tensor corresponding to one-hot encoded spike sequences of shape *(samples, sequence length, 4)*, where the four binary channels represent A, C, G, and T nucleotides. ReLU activations (Fred Agarap, 2018) were applied after each convolution to introduce non-linearity and mitigate vanishing gradients, allowing the model to learn complex feature hierarchies. MaxPooling1D layers were interleaved after each convolutional block to downsample the feature maps, reduce spatial dimensions, and control overfitting. Dropout layers with rates of 0.5, 0.2, and 0.1 were also inserted between layers for regularization (Srivastava et al., 2014). Padding was set to “same” to preserve spatial dimensions and prevent boundary effects during convolution operations (Gholamalinezhad and Khosravi, 2020) (Table 3).

**Table 3:** CNN–BiLSTM architecture for SARS-CoV-2 variant classification.

Step	Operation	Output dimension
Input Layer	One-Hot Encoding	3,854 × 13
Convolutional Layer 1	Conv1D (54 filters, size 6) + ReLU	3,849 × 54
	MaxPooling1D (pool = 5)	769 × 54
	Dropout (0.5)	769 × 54
Convolutional Layer 2	Conv1D (27 filters, size 3) + ReLU	769 × 27
	MaxPooling1D (pool = 3)	256 × 27
	Dropout (0.2)	256 × 27
Convolutional Layer 3	Conv1D (14 filters, size 2) + ReLU	256 × 14
	MaxPooling1D (pool = 3)	85 × 14
	Dropout (0.1)	85 × 14
Convolutional Layer 4	Conv1D (7 filters, size 2) + ReLU	85 × 7
	MaxPooling1D (pool = 3)	28 × 7
BiLSTM Layer	Bidirectional LSTM (512 units)	1,024
	Dropout (0.01)	1,024
Dense Layer 1	Dense (256 units) + ReLU	256
Dense Layer 2	Dense (128 units)	128
Dense Layer 3	Dense (64 units)	64
Dense Layer 4	Dense (32 units)	32
Dense Layer 5	Dense (16 units)	16
Output Layer	Dense (5 units) + Softmax	5 (classes)

Parameters	
Total parameters	2,445,805
Trainable parameters	2,445,805
Non-trainable	0

#### 2.5.2 BiLSTM layer and dense network

The feature map output from the last convolutional block was passed to a BiLSTM layer (512 units) to learn contextual nucleotide dependencies in both forward and reverse directions, critical for variant-discriminative patterns that may span across the sequence (Graves, 2012; Hochreiter and Schmidhuber, 1997). A dropout layer (0.01) followed the BiLSTM to prevent the co-adaptation of neurons. The high-level features were then flattened and passed through six fully connected Dense layers (256, 128, 64, 32, 16, and 5 units, respectively). All intermediate layers used ReLU activation, while the output layer employed a softmax function to predict the probability distribution across the five SARS-CoV-2 variant classes. The full architecture consisted of 2.4 million trainable parameters. The CNN–BiLSTM framework had a softmax output for multiclass classification, enabling fine-grained discrimination among SARS-CoV-2 variants. The softmax layer transforms the final outputs into probability distributions over variant classes (Wang et al., 2018), enabling clear and interpretable multiclass predictions. Crucially, this architecture not only improved performance on closely related lineages, particularly those with shared or overlapping mutation profiles but also facilitated interpretability via saliency mapping.

#### 2.5.3 Training configuration

The model was trained using the Adam optimizer (Kingma and Ba, 2014) with categorical cross-entropy as the loss function. One-hot encoded class labels were used to supervise training. The training dataset was split using stratified sampling (75% training, 25% test), and the validation set was held out to validate generalization. To enhance statistical reproducibility and consistency, we fixed random seeds across NumPy, TensorFlow, and Python environments (Bouthillier et al., 2021). Early stopping with patience of 8 epochs and a minimum delta of 0.0005 was applied to prevent overfitting and restore the best weights.

The model was trained for a maximum of 30 epochs using a batch size of 1,000. Epoch-wise accuracy and loss metrics were plotted for both training and validation datasets. We performed hyperparemeter tuning as the number of epochs increased, by adding more layers or removing some layers and while observing the model accuracy on model training. The model was trained and evaluated across 10 independent runs with different random initializations. All reported performance metrics represent the average values computed over these 10 runs to ensure robustness and mitigate variance due to stochastic processes during training.

#### 2.5.4 Model evaluation metrics and interpretation

Model performance was comprehensively evaluated using a multi-faceted approach designed to assess both predictive accuracy and interpretability. First, accuracy and loss metrics were recorded across training epochs to monitor convergence and generalization behavior. To provide a more granular understanding of classification performance, a detailed classification report was generated, including per-class precision, recall, and F1-scores, alongside macro-averaged metrics. Class-wise discrimination ability was further visualized through confusion matrices to identify misclassification patterns among SARS-CoV-2 variants. In addition, receiver operating characteristic (ROC) curves were constructed for each class, and the macro-average area under the curve (AUC) was computed to quantify the model’s ability to distinguish between variant classes (Fawcett, 2006). Beyond accuracy, the reliability of the model’s predicted probabilities was assessed through confidence calibration analysis. We calculated the Expected Calibration Error (ECE) and Maximum Calibration Error (MCE) using 10 equal-width confidence bins. A reliability diagram was plotted to illustrate the correspondence between predicted confidence and observed accuracy, and class-wise ECE values were reported to identify calibration discrepancies across variant types (Guo et al., 2017; Kull et al., 2019; Vaicenavicius et al., 2019). To enhance model interpretability, we applied gradient-based saliency map techniques which highlighted sequence positions with the strongest influence on classification decisions, revealing biologically meaningful nucleotide patterns and mutation hotspots. Reported saliency results were averaged over multiple runs to ensure robustness and reduce stochastic artifacts (Samek et al., 2021).

#### 2.5.5 Quantization for model optimization

While the CNN–BiLSTM model exhibited strong classification performance, its original architecture comprised approximately 2.45 million parameters, resulting in a memory footprint of ~9.3 MB. Such resource demands may hinder deployment in memory-constrained environments such as portable diagnostic devices, edge AI systems, or embedded viral surveillance platforms. To mitigate this limitation and enhance model portability, we applied post-training quantization using TensorFlow Lite (TFLite), a widely adopted framework for neural network compression and cross-platform deployment (David et al., 2021). Quantization was performed by converting the trained model from full precision (32-bit floating-point) to reduced precision using 8-bit integer weights while preserving float32 outputs. We employed dynamic range quantization via the TFLite converter, which statically quantizes only the model weights, thereby minimizing the need for representative datasets and preserving inference throughput (Jacob et al., 2018). To ensure compatibility with the BiLSTM architecture, we enabled SELECT_TF_OPS alongside TFLITE_BUILTINS, allowing the converter to fallback to TensorFlow operations when needed. Additionally, the experimental tensor list-lowering option was disabled to avoid incompatibility during the conversion of recurrent components.

### 2.6 Model validation

An external validation dataset, composed of SARS-CoV-2 spike sequences distinct from those used during training, was preprocessed using the same alignment, one-hot encoding, and dimensionality normalization procedures. This validation dataset contained sequences from all five target variants and was used to assess the model’s generalization to unseen data. Predictions were made using the trained CNN-BiLSTM quantized model, and performance was quantified using overall accuracy, per-class precision, recall, F1-score, and confusion matrices. The model maintained consistent classification accuracy, demonstrating its ability to generalize to new variant profiles beyond the training distribution.

### 2.7 Leave-One-Variant-Out cross-validation

To evaluate the robustness and generalization capacity of our CNN–BiLSTM classifier, we implemented a Leave-One-Variant-Out (LOVO) cross-validation scheme. This strategy simulates real-world scenarios where a previously unobserved variant emerges in the population. In each of the five LOVO experiments, one VOC was entirely excluded from the training dataset and reserved for validation. The model was trained on the remaining four variants, tested, and then validated. In addition to standard evaluation metrics (accuracy, precision, recall, F1-score, and confusion matrix), we employed confidence thresholding to flag predictions with low model certainty as “Unknown.” This mechanism enhances open-set recognition, allowing the model to abstain from forced classification when variant signatures deviate substantially from those learned during training (Fort et al., 2021; Qu et al., 2024).

## 3 Results and discussion

### 3.1 Dataset composition and model training overview

The curated Training and validation dataset comprised a total of 27,236 and 8,585 aligned spike protein DNA sequences, respectively, spanning five major SARS-CoV-2 variants (Figure 2). The relatively low number of Omicron sequences from GSAID database was due to their limited availability in the selected timeframe and stringent filtering criteria, hence enriched with NCBI Virus sequences. For supervised training, the dataset was partitioned into 20,427sequences (75%) for training and 6,809 sequences (25%) for testing, following a stratified sampling strategy to preserve variant class proportions. The final CNN–BiLSTM architecture contained approximately 2.45 million trainable parameters, reflecting the model’s depth and capacity for hierarchical feature learning. Each training session using a batch size of 1,000 and 30 epochs required approximately 65 min to converge using the Adam optimizer and early stopping based on validation accuracy.

### 3.2 Post-training quantization results

The quantized model demonstrated a substantial reduction in size and parameter count, from 2.45 million parameters (9.33 MB) to 465,997 parameters (3.95 MB), corresponding to an ~81% compression. Despite this reduction, the quantized model retained virtually identical classification performance on the test set and external validation dataset. Furthermore, inference time per sequence was reduced by 32% on CPU when evaluated in batch mode. This efficiency gain positions the model for practical deployment in real-time genomic surveillance systems or field diagnostics. These results further validate the suitability of post-training quantization as an effective model compression strategy for SARS-CoV-2 variant classification.

### 3.3 Model testing

**Figure 3:**
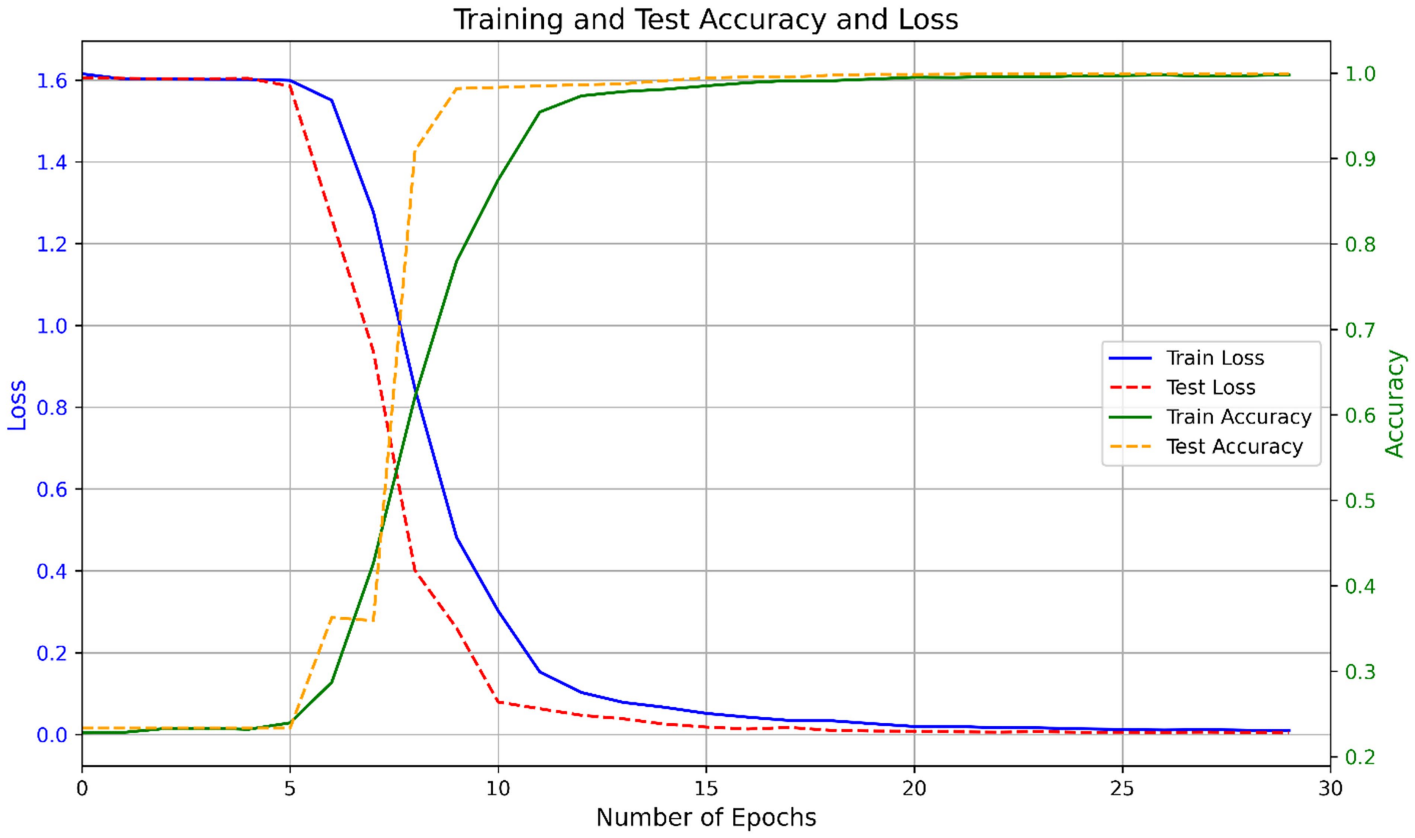
Training and test performance of the CNN-BiLSTM model across epochs.

To train the model, the one-hot encoded spike protein sequences were transformed into a tensor matrix and passed through a deep CNN–BiLSTM architecture. The training was guided by the Adam optimizer, with categorical cross-entropy as the loss function. Model weights were updated using backpropagation based on prediction error. The training process showed consistent convergence across multiple runs. On average, the model completed training in 25.9 ± 1.6 epochs. Initial training and testing accuracies were 0.2257 ± 0.0000 and 0.2335 ± 0.0000, respectively, with corresponding losses of 1.6311 ± 0.0000 and 1.6060 ± 0.0000, indicating randomized predictions at initialization. By the end of training, the model achieved a final training accuracy of 0.9974 ± 0.0011 and a test accuracy of 0.9991 ± 0.0003, with losses reduced to 0.0099 ± 0.0015 and 0.0037 ± 0.0006, respectively. Figure 3 shows the variation in accuracy and loss during training and testing.

**Figure 4:**
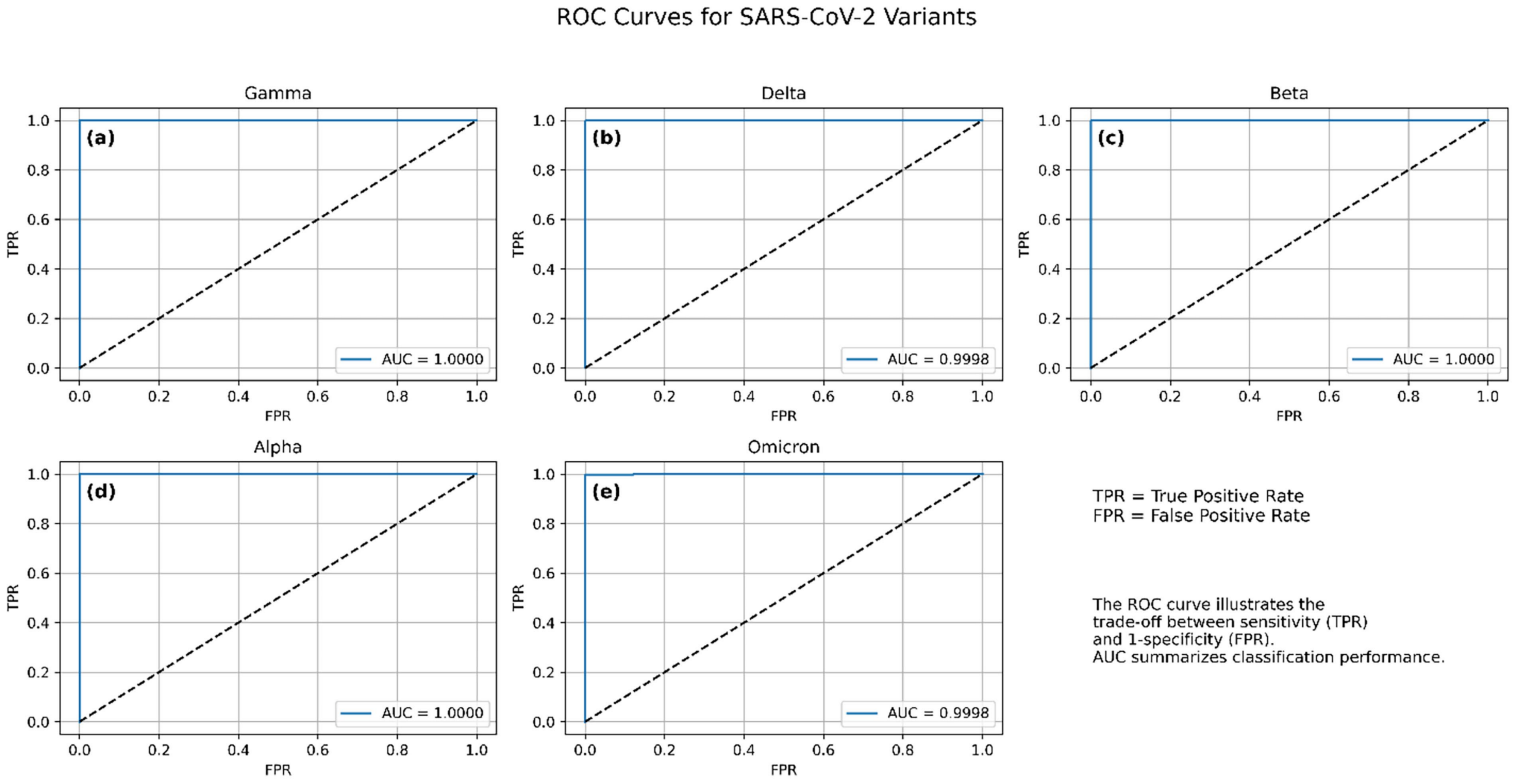
Receiver operating characteristic (ROC) curves for the CNN-BLSTM model across the five SARS-CoV-2 variant classes. **(a)** Gamma, **(b)** Delta, **(c)** Beta, **(d)** Alpha, and **(e)** Omicron. Each curve illustrates the trade-off between true positive rate (TPR) and false positive rate (FPR) for one variant, with all classes achieving near-perfect discrimination.

The ROC curve plots the true positive rate (sensitivity) against the false positive rate (specificity) at various classification thresholds, offering an interpretable measure of class separability. Across 10 independent training runs, the model consistently achieved near-perfect ROC-AUC values, indicating excellent generalization and class discrimination capabilities. The macro-average ROC-AUC was 0.9999 ± 0.00003, reflecting consistently high performance across all five variant classes. Individual variant-level AUC scores were equally strong: Alpha, Gamma, and Beta variants achieved perfect separability with AUC = 1.0000 ± 0.0000, while Delta and Omicron also demonstrated near-perfect scores of 0.9998 ± 0.00005, and 0.9998 ± 0.0002, respectively. These results confirm the model’s ability to robustly distinguish between SARS-CoV-2 variants using spike gene sequences. The small standard deviations across runs further demonstrate the stability and reproducibility of the architecture under different random initializations and training conditions. Visualizations of per-class ROC curves and macro-averaged curves are shown in Figure 4, which further illustrate the high separability achieved between variant classes.

An AUC of 1.00 represents an ideal classifier with 100% sensitivity and 100% specificity, which suggests that the model was able to flawlessly distinguish between all variant classes in the validation dataset. This exceptional discriminative power affirms that the model successfully learned robust and lineage-specific genomic features, making it highly effective in variant identification. Such performance is rarely achieved in genomic sequence classification, especially across closely related variants like Beta and Gamma, which share critical spike protein mutations such as E484K and N501Y (Harvey et al., 2021). Moreover, the distinctiveness of the Omicron variant, characterized by numerous spike protein mutations including P681H, K417N, and T478K, was captured by the model. The near-vertical ROC curve paths toward the upper-left corner reflect that the false positive rate was essentially zero across all classes. These results further corroborate the outcomes from the confusion matrix, where misclassifications were minimal and predominantly confined to variants with overlapping mutational landscapes. Such high AUC scores may also suggest that the model avoided overfitting due to its integrated architecture (CNN-BiLSTM), which is well-suited for capturing both local sequence motifs and long-range dependencies.

**Figure 5:**
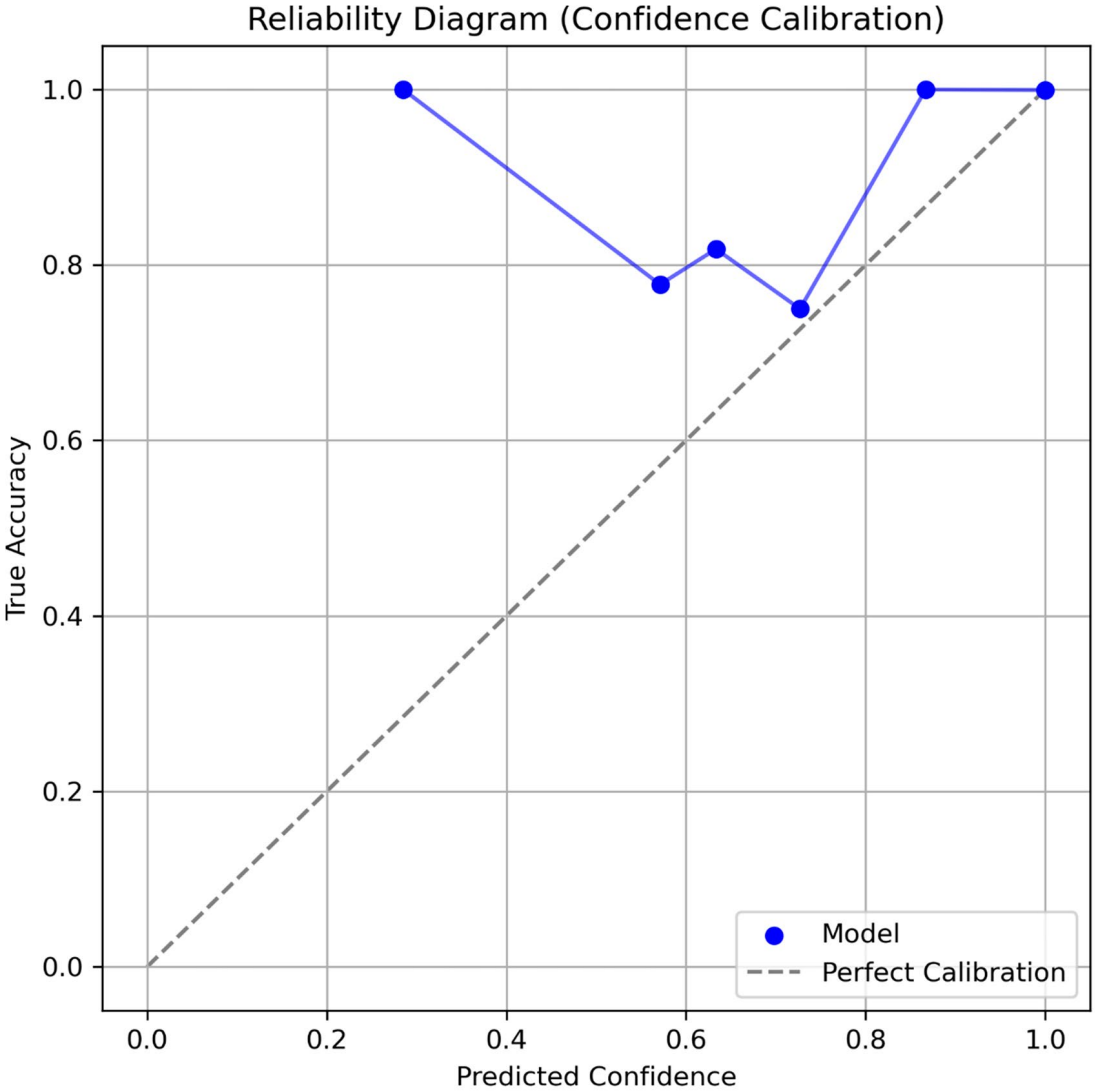
Reliability diagram showing confidence calibration of the CNN-BiLSTM model in classifying SARS-CoV-2 variants using spike gene sequences. The blue curve represents model confidence vs. actual accuracy, and the dashed diagonal line indicates perfect calibration. A model above the perfect calibration shows a good calibration.

The calibration analysis revealed that the model provides highly reliable confidence scores (Figure 5). This was visualized through a reliability diagram, which shows close alignment with the ideal calibration line. The expected calibration error (ECE) for the overall model was extremely low at 0.0009 ± 0.0003, indicating that the predicted probabilities closely match actual accuracies. Additionally, the maximum calibration error (MCE) was 0.1659 ± 0.0776, a tolerable deviation in low-confidence bins. Calibration was also evaluated per variant: Gamma (ECE = 0.0001 ± 0.0000), Delta (0.0006 ± 0.0001), Beta (0.0002 ± 0.0001), Alpha (0.0005 ± 0.0001), and Omicron (0.0005 ± 0.0002). These values show consistent calibration performance across different SARS-CoV-2 lineages. The model demonstrates low expected calibration error, indicating trustworthy confidence estimates.

**Figure 6:**
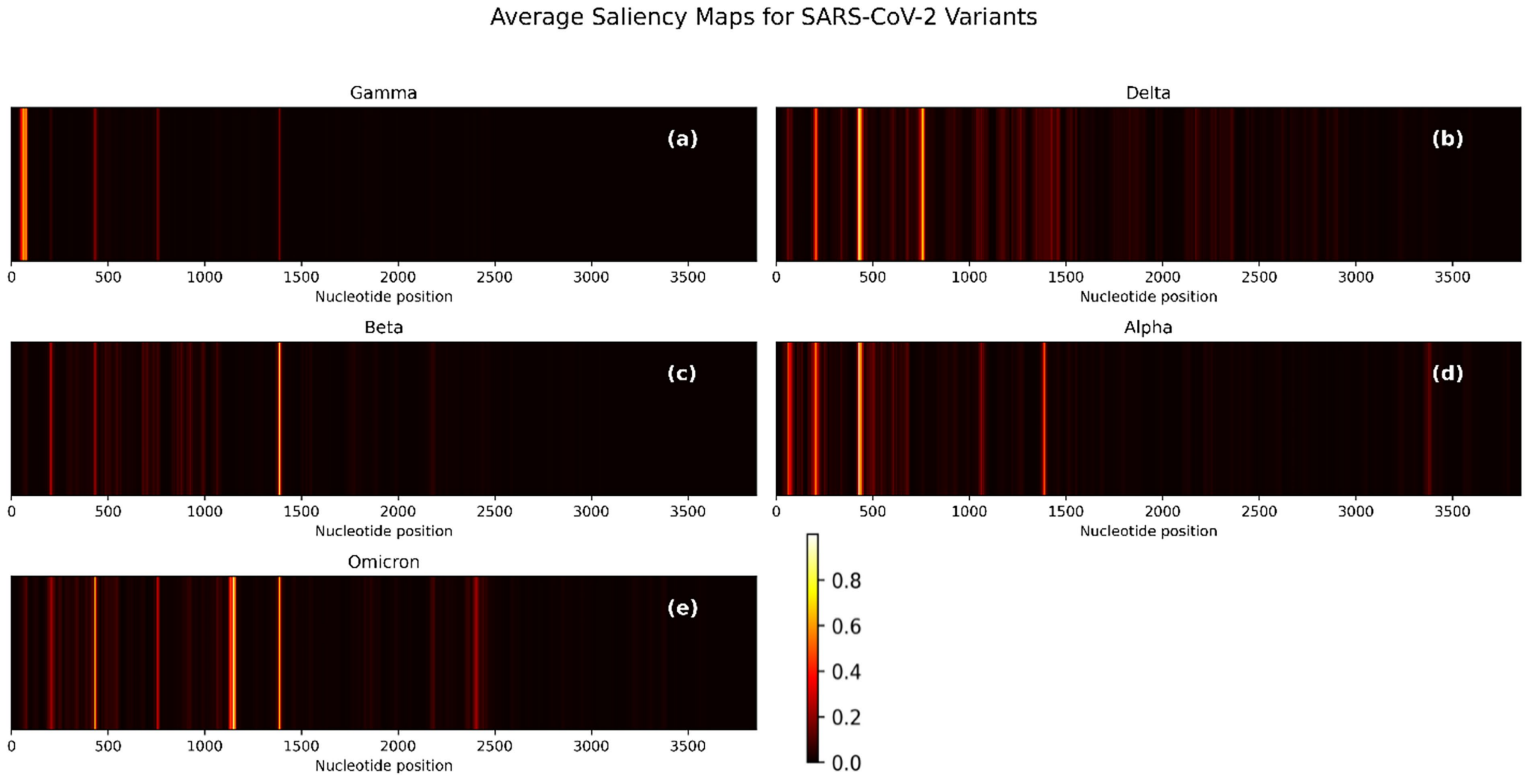
Average saliency maps across nucleotide positions for five SARS-CoV-2 variants based on spike gene input. **(a)** Gamma, **(b)** Delta, **(c)** Beta, **(d)** Alpha, and **(e)** Omicron. Bright regions indicate higher model sensitivity and importance. The intensity of each signal correlates with the feature’s importance to classification.

To evaluate the interpretability of the CNN-BiLSTM model, we generated pre-quantization saliency maps for each SARS-CoV-2 variant by averaging gradient-based importance scores across nucleotide positions in the spike gene. These visualizations (Figure 6) reveal how the model identifies critical regions responsible for classification. The saliency maps for each variant uncover biologically relevant patterns that correspond to known mutational hotspots and sequence patterns in the spike gene, which support variant discrimination. To zoom through these maps, the precise nucleotide positions whose saliency values are above 0.2 were extracted for each variant and converted into saliency plots (Supplementary Figures S8–S12).

The saliency maps (Figure 6; Supplementary Figures S8–S12) reveal distinct nucleotide patterns prioritized by the CNN-BiLSTM classification model across SARS-CoV-2 variants. For Gamma (Figure 6a; Supplementary Figure S1), the most prominent signal arises from nucleotides 55–58 (CAAA), indicating a concentrated contribution to model prediction in the 5′ region of the spike gene. Aside from a notable position at nucleotide 1,386, the majority of salient features lie below position 100, suggesting the presence of a contiguous informative region between positions 46–82 rather than isolated single-nucleotide contributions. In the Delta variant (Figure 6b; Supplementary Figure S9), the saliency signal is broader and denser, with recurrent patterns rather than singular positions dominating the model’s focus. Salient regions include 197–213, 421–449, and 735–766. Additionally, discrete peaks at positions 1,413, 1,428, 1,454, and 1,461 further underscore the variant-specific features influence distributed across the spike sequence.

For Beta (Figure 6c; Supplementary Figure S10), salient features are highly localized, with a concentrated peak spanning positions 1,379–1,394. Secondary signals are observed at positions 431 and 202–207, indicating a narrower mutational signature influencing the model’s classification. The Alpha variant (Figure 6d; Supplementary Figure S11) exhibits distinct saliency around positions 194–217, a region potentially corresponding to the Δ69–70 deletion, a known defining mutation of Alpha. Beyond this, the model appears to prioritize diffuse patterns over discrete mutation sites, suggesting its reliance on broader sequence motifs. Omicron (Figure 6e; Supplementary Figure S12) displays an expansive and intensified saliency profile, with multiple peaks across the spike gene, particularly at 201–208, 429–437, 753–762, 1,125–1,159, 1,379–1,392, and several positions beyond nucleotide 2000. This widespread attention aligns with Omicron’s extensive mutational load, which includes over 30 spike mutations (Karim and Karim, 2021) and reflects the model’s capacity to capture complex, lineage-specific patterns. Notably, a conserved saliency signal is consistently observed across all variants in the 0–500 nucleotide range. Although these regions may not correspond to well-characterized mutations, they likely reflect conserved sequence motifs or alignment-related artifacts that the model has learned to associate with class-discriminative features. This warrants further interpretability-focused investigations to elucidate the biological or technical underpinnings of these recurrent signals. These results confirm that the one-hot encoding approach does not preclude the model from capturing complex contextual dependencies. Despite lacking explicit k-mer context, the CNN–BiLSTM architecture effectively identified multi-base sequence motifs relevant to variant classification. Notably, high saliency regions often spanned multiple positions and exhibited clear lineage-specific patterns, reinforcing the biological plausibility of our learned representations.

### 3.4 Model validation

Following successful training and testing of the model, further validation of the model was performed on a new dataset containing 8,585 SARS-CoV-2 variant sequences downloaded from the GSAID (3,062) and NCBI virus (5,523) databases. The distribution of variants in the validation dataset is shown in Figure 2b.

**Figure 7:**
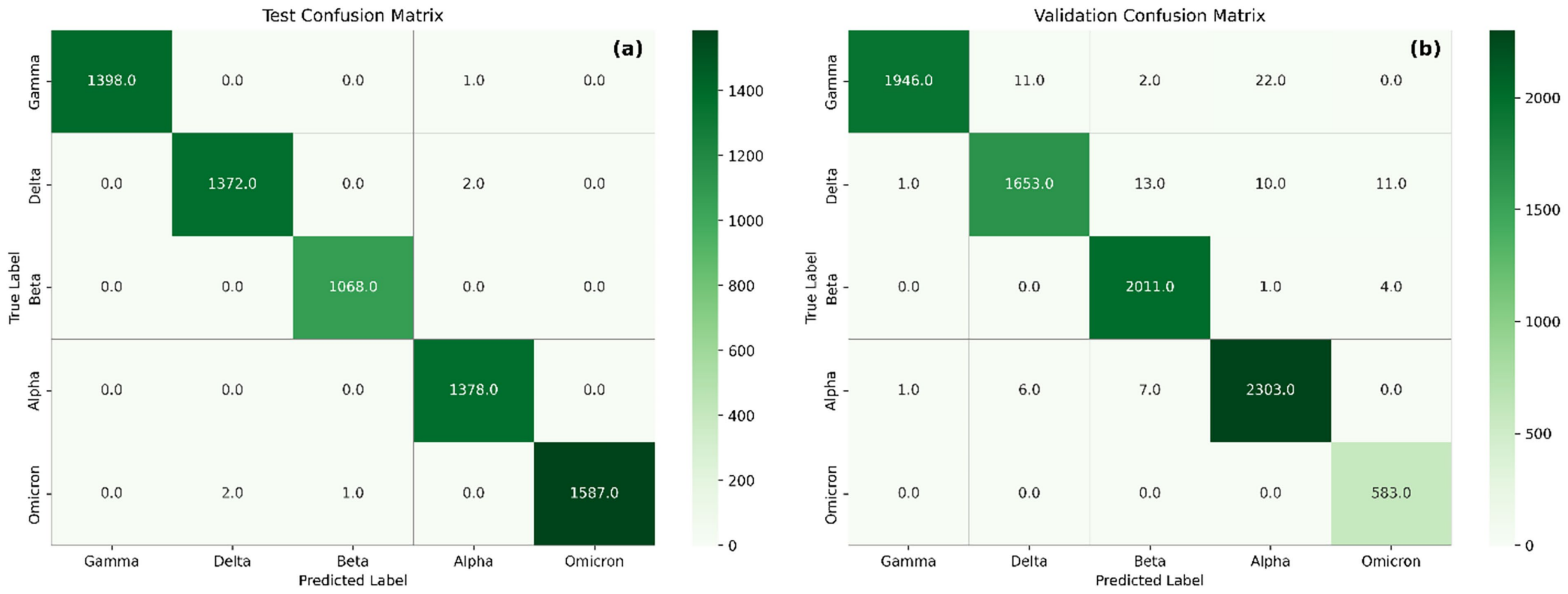
Confusion matrix showing the performance of the model on the test set **(a)** and validation set **(b)**.

Additionally, confusion matrices (Figure 7) were used to assess per-class performance and misclassification patterns in both test and external validation datasets. Confusion matrices show strong agreement between true and predicted labels, with nearly all variants correctly classified in both test and validation sets. In the test matrix (Figure 7a), classification accuracy exceeds 99% across all variants, with negligible confusion.

In the external validation matrix, most variants demonstrate high classification accuracy, reflecting the model’s robustness. Nonetheless, several misclassifications are observed. Delta is occasionally misclassified as Beta (*n* = 13), suggesting overlapping feature representations, which is further supported by similarities observed in their saliency maps. Alpha misclassified as Beta (*n* = 7) may result from shared sequence features that contribute to model decisions, particularly within regions around position 1,500 and below 500. Although limited in number, Gamma misclassified as Beta (*n* = 2) likely stems from both variants sharing critical receptor-binding domain (RBD) mutations such as E484K and N501Y, highlighting the difficulty in distinguishing Gamma from Beta based solely on RBD signatures. Additional misclassifications include Gamma to Alpha (*n* = 22) and Delta to Alpha (*n* = 10), Delta to Omicron (*n* = 11), Alpha to Delta (*n* = 6), Alpha to Gamma (*n* = 1), Beta to Alpha (*n* = 1), and Beta to Omicron (*n* = 4). Importantly, all 583 Omicron sequences were correctly classified, demonstrating the model’s strong generalization capability for highly mutated variants when included in training. This likely arises from Omicron’s distinct profile, which includes shared mutations with multiple variants (e.g., N501Y, Δ69–70), but also unique substitutions such as N679K and multiple insertions that provide discriminative power (Cao et al., 2021; Karim and Karim, 2021).

This study included a confusion report for the validation dataset that has the precision, recall, accuracy, and F1 score for the 5 classes, shown in Table 4. Precision is the ability of the model to predict the true positive sequence as positive, and recall shows the number of positive sequences that are classified correctly as positive. The F1 scoresums up the predictive performance of the model by combining precision and recall metrics. It gives the overall accuracy of the model. This is calculated from the precision and recall test as shown in the following formulas.


$$\text{Precision}=\frac{\text{True positive}}{\text{True positive}+\text{False Positive}}$$

$$\text{Recall}=\frac{\text{True positive}}{\text{True Positive}+\text{Negative}}$$

$$F1-\text{Score}=\frac{2^{*}\;{\text{Precision}}^{*}\;\text{Recall}}{\text{Precision}+\text{Recall}}$$

**Table 4:** Classification performance of the CNN-BiLSTM model on the external validation dataset across five SARS-CoV-2 variants.

Variant	Precision (%)	Recall (%)	F1-score (%)	Support
Gamma	100.00 ± 0.00	98.00 ± 0.00	99.00 ± 0.00	1981
Delta	99.00 ± 0.00	98.00 ± 0.30	98.00 ± 0.00	1,688
Beta	99.00 ± 0.10	100.00 ± 0.00	99.00 ± 0.00	2016
Alpha	99.00 ± 0.00	99.00 ± 0.13	99.00 ± 0.00	2,317
Omicron	97.00 ± 0.10	100 ± 0.10	99.80 ± 0.13	583

Metric	Mean ± SD (%)	Support
Accuracy	99.00 ± 0.00	8,585
Macro Avg Precision	99.16 ± 0.08	8,585
Macro Avg Recall	99.14 ± 0.12	8,585
Macro Avg F1-score	99.16 ± 0.08	8,585
Weighted Avg Precision	99.00 ± 0.00	8,585
Weighted Avg Recall	99.00 ± 0.00	8,585
Weighted Avg F1-score	99.00 ± 0.00	8,585

Metrics are reported as mean ± standard deviation over 10 independent runs. Precision, recall, and F1-score are expressed as percentages.

The model demonstrated exceptional classification performance on the external validation dataset, achieving an overall accuracy of 99.00% ± 0.00 across five SARS-CoV-2 variants. Precision, recall, and F1scores were consistently high across all classes, with minimal variance across runs, indicating strong robustness and generalizability of the model. Per-class analysis reveals the model’s ability to accurately distinguish between closely related viral lineages. Gamma variant was predicted with perfect precision (100.00 ± 0.00%), while beta, delta, and alpha with a precision of 99.00 ± 00% while omicron had the smallest of 97.00 ± 00% with all their recall ≥98.00 ± 00%, underscoring the model’s sensitivity to discriminative mutation patterns within these classes. The Omicron variant, which is of particular concern due to its rapid global spread and mutational complexity, was identified with 99.00 ± 0.13% F1-score, reflecting high sensitivity (recall: 99.00 ± 0.10%) and specificity (precision: 97.00 ± 0.10%), even with limited support (*n* = 583). This is noteworthy given the evolutionary divergence of Omicron from other lineages and the model’s training being predominantly on earlier variants. The Beta variant, known for its immune escape features, showed a higher precision (99.00 ± 0.10%) and maintained 100 ± 0.00% recall, suggesting that the model prioritizes avoiding false negatives, a desirable trait in surveillance applications. Delta, with a recall of 98.10% ± 0.30, showed the lowest among the five, though still indicative of high performance. Macro-averaged and weighted precision, recall, and F1-scores remained consistently 99.00%, highlighting the model’s balanced performance regardless of minimal class imbalance. This confirms the model’s strong generalization ability and aligns with findings in genomic deep learning, where CNN-RNN hybrids outperform traditional ML classifiers in sequence-based virus classification tasks (Randhawa et al., 2020; Zeng et al., 2016).

These results suggest that the CNN–BiLSTM architecture can reliably classify SARS-CoV-2 variants from raw nucleotide sequences, offering a rapid and alignment-free computational tool suitable for genomic surveillance, especially in settings where fast mutation tracking is critical. Furthermore, the consistency of performance across 10 independent runs (reported as mean ± standard deviation) provides statistical robustness in line with best practices in modern deep learning evaluation (Charles et al., 2023; Charles and Mahapatra, 2023; Charles and Edgar, 2024; Reimers and Gurevych, 2017). The model demonstrates consistently high performance across all variants, with minimal variability, indicating robustness and strong generalization capacity.

We compared the validation results from our model with the classification results from Nextclade, a genomic analysis tool used for SARS-CoV-2 variant assignment (Aksamentov et al., 2021; Hadfield et al., 2018). Table 5 presents the performance metrics for both tools. The CNN + BiLSTM model demonstrated strong performance, achieving over 95% accuracy for all VOCs. Notably, the model excelled in classifying Omicron, with a perfect success rate of 100%, outperforming Nextclade’s 34.95% for this variant. Nextclade showed high accuracy (98–99%) for most variants, including Alpha, Beta, Gamma, and Delta, but struggled significantly with Omicron, correctly classifying only 34.95% of sequences. Additionally, Nextclade identified the presence of recombinant strains in Omicron (*n* = 67) and Delta (*n* = 11), a capability that is currently outside the scope of our CNN + BiLSTM model. While both tools perform well for most variants, Nextclade’s recombinant strain identification adds value to its analysis, but its limitations in Omicron classification underscore the strength of our CNN + BiLSTM model in this area.

**Table 5:** Comparison between the proposed model and the Nextclade in variant classification.

Variant	Total	Nextclade				CNN + LSTM Model	
		Assigned	%	Unassigned	Recombinant	Assigned	Model success %
Alpha	625	620	99.20	5	0	615	98.40 ± 0.00
Beta	1,403	1,398	99.64	5	0	1,398	99.64 ± 0.02
Gamma	437	436	99.77	1	0	432	98.86 ± 0.00
Delta	494	482	97.57	12	11	472	95.55 ± 0.50
Omicron	103	36	34.95	0	67	103	100 ± 0.00

One-hot encoding and a k-mer feature classification models (k = 3) were also constructed to benchmark our model, and their performance was compared to our CNN-BiLSTM model. The results show that our model is superior to these models. Table 6 summarizes the comparison.

**Table 6:** Comparison of our model to baseline models.

Feature representation method	Method	Acc.	Recall	F1 (Weig.)	F1 (Macro)	Prec.
k-mer	SVM	0.36	0.39	0.25	0.26	0.22
	DT	0.36	0.39	0.20	0.22	0.15
	MLP	0.36	0.39	0.25	0.26	0.23
	KNN	0.36	0.40	0.24	0.25	0.22
	RF	0.36	0.40	0.23	0.24	0.20
	RC	0.37	0.40	0.24	0.25	0.21
	LR	0.36	0.39	0.24	0.25	0.22
	NB	0.36	0.40	0.23	0.27	0.23
OHE	SVM	0.37	0.40	0.26	0.27	0.24
	DT	0.37	0.40	0.22	0.23	0.18
	MLP	0.37	0.40	0.26	0.27	0.24
	KNN	0.37	0.40	0.25	0.26	0.23
	RF	0.143	0.20	0.04	0.05	0.03
	RC	0.37	0.40	0.25	0.26	0.22
	LR	0.37	0.40	0.25	0.26	0.22
	NB	0.36	0.39	0.23	0.25	0.22
CNN-BiLSTM	**CNN-BiLSTM**	**0.99 ± 0.00**	**0.98 ± 0.00**	**0.99 ± 0.00**	**0.99 ± 0.00**	**0.99 ± 0.00**

The superior values are shown in bold.

### 3.5 LOVO

A confidence threshold of 0.603 was empirically selected based on calibration curves (see Supplementary Figure S2). When Alpha was left out during training, the model failed to identify this class entirely, yielding an F1-score of 0.00 (Supplementary Table S2). The majority of Alpha sequences with over 96% were flagged as “Unknown,” (Supplementary Figure S3). This reflects the limitations of closed-set classifiers under open-set conditions, particularly when overlapping RBD mutations such as N501Y (shared with Beta, Gamma, and Omicron) mislead the network. In the Beta-excluded setup, the classifier showed complete failure in Beta recognition (F1 = 0.00), with misclassifications skewed toward Delta and Alpha (Supplementary Table S3), This is not a surprise as Beta and Delta share the main important 3 saliency features, even though they are more significate for Delta than Beta (Figures 6b,c). Notably, a few numbers of sequences were also labeled as “Unknown,” reflecting the network’s uncertainty in the absence of Beta’s hallmark features (Figure 6c). The overall accuracy remained high (83%), but macro F1 was reduced to 0.65 (Supplementary Table S3).

Exclusion of Delta during training led to severe misclassification, with most Delta sequences being labeled as Beta/Omicron or flagged as “Unknown” (Supplementary Table S4; Supplementary Figure S5). While Gamma, Alpha, and Omicron maintained high classification fidelity (F1 > 0.97), the omission of Delta resulted in a macro F1 of 0.65, underlining the challenge of identifying this variant without exposure. Gamma sequences were also poorly classified when excluded from training, achieving an F1-score of 0.00 (Supplementary Table S5), and 99% were flagged as unknown (Supplementary Figure S6). Despite the lack of Gamma-specific representation during training, the classifier maintained high weighted accuracy (84%) due to strong performance on known variants. The omission of Omicron caused misclassification primarily into mainly Alpha, with a subset flagged as “Unknown” (Supplementary Figure S7; Supplementary Table S6). The divergence of Omicron’s mutational profile including more than 30 substitutions resulted in a complete failure to identify it correctly (F1 = 0.00), though the model maintained an overall accuracy of 95% on known classes.

The LOVO experiments reveal critical insights into the model’s generalization capacity and its behavior under open-set conditions. While the CNN-BiLSTM architecture demonstrated near-perfect classification on known classes, it struggled to generalize to previously unseen variants. Most failures were biologically grounded misclassification patterns aligned with known shared mutations partly supporting the interpretability of deep learning outputs.

Importantly, the use of confidence thresholding enabled a safety mechanism to flag low-confidence predictions. In the Alpha-left-out setup, for example, a majority of sequences were flagged as “Unknown” rather than being misclassified, reducing the risk of erroneous inference. This behavior reflects real-world utility, where genomic surveillance systems must handle emerging lineages conservatively before integration into training sets. The inclusion of the “Unknown” class also highlights the importance of uncertainty-aware classification in bioinformatics. Classical classifiers tend to force all predictions into known classes, which can mislead downstream interpretation. Our thresholding strategy enhances reliability by enabling abstention from classification when evidence is insufficient. Overall, LOVO testing provides a rigorous stress test for variant classifiers. Our findings suggest that while CNN–BiLSTM models offer superior performance when trained comprehensively, their application in real-time pathogen surveillance requires dynamic retraining and incorporation of model calibration mechanisms to ensure accurate performance in evolving genomic landscapes (Fort et al., 2021; Qu et al., 2024).

In this study, we trained and evaluated a CNN–LSTM deep learning model for classifying SARS-CoV-2 variants using spike gene nucleotide sequences. Traditional approaches, particularly k-mer-based and kernel approximation methods, have been widely employed for viral sequence analysis due to their computational efficiency and interpretability. These models generally perform well when sequence patterns are localized and relatively simple to capture through fixed-length representations (Ali et al., 2021). However, their reliance on predefined features restricts adaptability in dynamic genomic contexts. As SARS-CoV-2 continues to evolve, these models require frequent recalibration to accommodate novel mutations, limiting their utility for prospective surveillance applications. The choice of k-mer size further complicates their effectiveness: shorter k-mers may overlook long-range biological dependencies, while longer k-mers increase dimensionality and risk overfitting. Moreover, such methods cannot automatically discover meaningful hierarchical features or integrate distant relationships between sequence elements, and these limitations become increasingly problematic as genomic datasets grow in size and complexity (Ali et al., 2021; Ali and Patterson, 2021).

DL architectures, particularly hybrid models like CNN–LSTM, overcome these constraints by learning discriminative features directly from raw nucleotide sequences. CNN layers are well-suited for capturing spatial patterns such as conserved motifs or mutation clusters, while LSTM layers excel at modeling temporal dependencies, including long-range interactions between dispersed sequence elements (Graves, 2012; Hochreiter and Schmidhuber, 1997; Zhang et al., 2015). This layered architecture enables our model to learn both local and global sequence relationships without manual feature engineering, providing superior flexibility and generalization performance (Lecun et al., 2015; Ren et al., 2017). Our CNN–LSTM classifier demonstrated excellent accuracy (99%) on the independent validation dataset, highlighting its robustness in generalizing to unseen sequences. Importantly, the model was explicitly designed to tolerate real-world sequencing errors, including ambiguous nucleotides (“N”), which were ignored during training and prediction. By assigning zero weight to these bases, the model focused exclusively on informative regions of the sequence, enhancing reliability in noisy, high-throughput sequencing contexts (Juan et al., 2017).

The model’s performance also benefited from leveraging the spike gene, a functionally critical and mutation-dense region of the SARS-CoV-2 genome as the input feature space. Although this region represents only ~13% of the complete genome, it harbors key variant-defining mutations, making it a highly informative target for classification tasks (Harvey et al., 2021; Karim and Karim, 2021). Compared to rule-based molecular epidemiology tools like Nextclade, our approach showed superior accuracy, especially for divergent lineages such as Omicron, which are frequently misclassified due to their extensive mutational drift (Aksamentov et al., 2021).

To prevent overfitting and promote generalization, we implemented several regularization techniques. Dropout (Srivastava et al., 2014) was used to randomly deactivate neurons during training, reducing co-adaptation and improving robustness. L2 weight regularization (Goodfellow et al., 2016) penalized model complexity, and batch normalization accelerated convergence and stabilized training. These methods were complemented by 5-fold cross-validation to ensure performance consistency across diverse data splits, in line with best practices for evaluating deep learning models (Neyshabur et al., 2017). The use of the Adam optimizer further facilitated efficient convergence by dynamically adapting learning rates based on gradient estimates (Kingma and Ba, 2014). Compared to earlier works, our model offers a balanced solution to both interpretability and performance. For example, Whata and Chimedza (2021) proposed a CNN–BiLSTM model for broad coronavirus classification with excellent results, but limited computational depth constrained its scalability. Similarly, KEVOLVE (Lebatteux et al., 2024), which relies on genetic algorithms to extract discriminative motifs, demonstrated strong performance but may face interpretability and generalization challenges as new variants emerge. In our implementation, the CNN–LSTM model combines high accuracy, resilience to sequence ambiguity, and scalability, making it well-suited for real-time genomic surveillance.

## 4 Limitations and future work

Although the CNN–BiLSTM model developed in this study demonstrated high accuracy in classifying SARS-CoV-2 variants based on spike gene sequences, several limitations warrant careful consideration. First, the use of spike gene sequences as the sole input feature. Although the spike protein is biologically informative and central to variant-specific phenotypes such as transmissibility and immune escape, relying exclusively on this region may restrict the model’s ability to detect lineages that harbor distinguishing mutations elsewhere in the genome. The emergence of recombinant lineages or divergent subvariants with mutations outside the spike region could reduce classification performance. Expanding the input space to include full-genome sequences or other conserved regions could enhance the model’s generalizability to a broader array of SARS-CoV-2 lineages.

A further consideration is ambiguous nucleotides are treated as uninformative zeros, potentially excluding useful information in noisy real-world sequences. Alternative sequence encoding strategies such as transformer-based representations, or positional attention mechanisms could enable the model to learn context-dependent relationships and dynamically assign importance to specific regions of the spike gene.

The LOVO validation strategy exposed additional limitations in model generalization. Although the model maintained high accuracy when all classes were seen during training, performance deteriorated substantially when a variant was excluded, reflecting the difficulty in generalizing to previously unseen classes despite shared mutational features, revealing that the model’s decision boundaries were highly dependent on variant-specific training data. These findings underscore the challenge of open-set classification in genomics, where the ability to flag novel or recombinant lineages without prior exposure is crucial. Incorporating confidence calibration techniques and open-set recognition mechanisms may improve the model’s capacity to handle such cases.

In future iterations, the model will be extended to address these limitations, with a focus on improving efficiency, scalability, and adaptability. This will include the integration of unsupervised anomaly detection for early identification of novel variants and the benchmarking of CNN–BiLSTM against emerging architectures such as transformers. These enhancements aim to position the model as a robust and interpretable framework for genomic surveillance, capable of supporting variant classification in fast-evolving epidemiological landscapes.

## 5 Conclusion

This study shows that deep learning can be applied as an alternative method to the classification of viruses in addition to conventional sequence classification methods. CNN-LSTM model achieved high accuracy in classifying the five most dominant SARS-CoV-2 variants. Clinicians and public health organizations could use this tool to track new SARS-CoV-2 lineages in real-time surveillance settings. Frontline diagnostic workflows might benefit from its low latency and excellent precision.

## Data Availability

Publicly available datasets were analyzed in this study. This data can be found here: https://github.com/omicscodeathon/ml_sarscov2.

## References

[ref1] Abd-Alhalem S. M. El-Rabaie E.-S. M. Soliman N. F. Abdulrahman S. E. S. E. Ismail N. A. Abd El-samie F. E. (2021). DNA sequences classification with deep learning: a survey. Menoufia J. Electron. Eng. Res. 30, 41–51. doi: 10.21608/MJEER.2021.146090

[ref2] Aksamentov I. Roemer C. Hodcroft E. Neher R. (2021). Nextclade: clade assignment, mutation calling and quality control for viral genomes. J. Open Source Softw. 6:3773. doi: 10.21105/JOSS.03773

[ref3] Alaya F. Baraket G. Adediran D. A. Cuttler K. Ajiboye I. Kivumbi M. T.. (2024). Multiple sclerosis stages and their differentially expressed genes: a bioinformatics analysis. bioRxiv. doi: 10.1101/2024.01.20.576448

[ref4] Ali S. Bello B. Chourasia P. Punathil R. T. Zhou Y. Patterson M. (2022) PWM2Vec: an efficient embedding approach for viral host specification from coronavirus spike sequences. Available online at: http://arxiv.org/abs/2201.02273. 10.3390/biology11030418 PMC8945605 35336792

[ref5] Ali S. Bello B. Tayebi Z. Patterson M. (2023). Characterizing SARS-CoV-2 spike sequences based on geographical location. J. Comput. Biol. 30, 432–445. doi: 10.1089/CMB.2022.0391, PMID: 36656554

[ref6] Ali S. Patterson M. (2021). *Spike2Vec:* an efficient and scalable embedding approach for COVID-19 spike sequences. Available online at: http://arxiv.org/abs/2109.05019.

[ref7] Ali S. Sahoo B. Ullah N. Zelikovskiy A. Patterson M. Khan I. (2021). A k-mer based approach for SARS-CoV-2 variant identification. Available online at: http://arxiv.org/abs/2108.03465.

[ref8] Ather S. H. Awe O. I. Butler T. J. Denka T. Semick S. A. Tang W.. (2018). SeqAcademy: an educational pipeline for RNA-Seq and ChIP-Seq analysis. F1000Res 7:628. doi: 10.12688/f1000research.14880.4, PMID: 33014338 PMC7525341

[ref9] Awe O. I. En najih N. Nyamari M. N. Mukanga L. B. (2023). Comparative study between molecular and genetic evolutionary analysis tools using African SARS-CoV-2 variants. Inform. Med. Unlocked 36:101143. doi: 10.1016/j.imu.2022.101143

[ref10] Ben Aribi H. Abassi N. Awe O. I. (2024). Neurovar: an open-source tool for the visualization of gene expression and variation data for biomarkers of neurological diseases. Gigabyte 2024:gigabyte143-0. doi: 10.46471/gigabyte.143, PMID: 39629064 PMC11612633

[ref11] Ben Aribi H. Dixon I. Abassi N. Awe O. I. (2025). Efficient and easy gene expression and genetic variation data analysis and visualization using exvar. Sci. Rep. doi: 10.1038/s41598-025-93067-5 PMC11985497 40210898

[ref12] Bouthillier X. Delaunay P. Bronzi M. Trofimov A. Nichyporuk B. Szeto J.. (2021). Accounting for variance in machine learning benchmarks. Proceedings of Machine Learning and Systems. Available online at: https://proceedings.mlsys.org/paper_files/paper/2021/hash/0184b0cd3cfb185989f858a1d9f5c1eb-Abstract.html.

[ref13] Camacho C. Coulouris G. Avagyan V. Ma N. Papadopoulos J. Bealer K.. (2009). BLAST+: architecture and applications. BMC Bioinform. 10:421. doi: 10.1186/1471-2105-10-421, PMID: 20003500 PMC2803857

[ref14] Cao Y. Wang J. Jian F. Xiao T. Song W. Yisimayi A.. (2021). Omicron escapes the majority of existing SARS-CoV-2 neutralizing antibodies. Nature 602, 657–663. doi: 10.1038/s41586-021-04385-3, PMID: 35016194 PMC8866119

[ref15] Charles S. Edgar P. M. (2024). *Geometric deep learning* prioritization and validation of cannabis phytochemicals as anti-HCV non- nucleoside direct-acting inhibitors. Available online at: 10.21203/RS.3.RS-3961716/V1. PMC11638990 39678171

[ref16] Charles S. Edgar M. P. Mahapatra R. K. (2023). Artificial intelligence based virtual screening study for competitive and allosteric inhibitors of the SARS-CoV-2 main protease. J. Biomol. Struct. Dyn. 41, 15286–15304. doi: 10.1080/07391102.2023.2188419, PMID: 36943715

[ref17] Charles S. Mahapatra R. K. (2023). Artificial intelligence based de-novo design for novel *Plasmodium falciparum* plasmepsin (PM) X inhibitors. J. Biomol. Struct. Dyn. 43, 92–107. doi: 10.1080/07391102.2023.2279700 37943000

[ref18] Coetzer K. C. Zemzem F. Akurut E. Wiafe G. A. Awe O. I. (2025). RareInsight simplifies the communication of genetic results for rare disease patients. Sci. Rep. 15:24442. doi: 10.1038/s41598-025-09744-y, PMID: 40628872 PMC12238553

[ref19] David R. Duke J. Jain A. Reddi V. J. Jeffries N. Li J.. (2021). Tensorflow lite micro: embedded machine learning for tinyml systems. Proceedings of machine learning and systems. Available online at: https://proceedings.mlsys.org/paper_files/paper/2021/hash/6c44dc73014d66ba49b28d483a8f8b0d-Abstract.html.

[ref20] Die J. V. Elmassry M. M. LeBlanc K. H. Awe O. I. Dillman A. Busby B. (2019). geneHummus: an R package to define gene families and their expression in legumes and beyond. BMC Genomics 20:591. doi: 10.1186/s12864-019-5952-2, PMID: 31319791 PMC6639926

[ref21] Edara V. V. Norwood C. Floyd K. Lai L. Davis-Gardner M. E. Hudson W. H.. (2021). Infection- and vaccine-induced antibody binding and neutralization of the B.1.351 SARS-CoV-2 variant. Cell Host Microbe 29, 516–521.e3. doi: 10.1016/j.chom.2021.03.009 33798491 PMC7980225

[ref22] El Abed F. Baraket G. Nyamari M. N. Naitore C. Awe O. I. (2023). Differential expression analysis of miRNAs and mRNAs in epilepsy uncovers potential biomarkers. bioRxiv. doi: 10.1101/2023.09.11.557132

[ref23] Elbasir A. Ye Y. Schäffer D. E. Hao X. Wickramasinghe J. Tsingas K.. (2023). A deep learning approach reveals unexplored landscape of viral expression in cancer. Nat. Commun. 14, 785–712. doi: 10.1038/s41467-023-36336-z, PMID: 36774364 PMC9922274

[ref24] Enejoh O. A. Okonkwo C. H. Nortey H. Kemiki O. A. Ainembabazi M. Mbaoji F. N.. (2025). Machine learning and molecular dynamics simulations predict potential TGR5 agonists for type 2 diabetes treatment. Front. Chem. doi: 10.3389/fchem.2024.1503593 PMC11754275 39850718

[ref25] Fawcett T. (2006). An introduction to ROC analysis. Pattern Recogn. Lett. 27, 861–874. doi: 10.1016/J.PATREC.2005.10.010

[ref26] Fort S. Ren J. Lakshminarayanan B. (2021) Exploring the limits of out-of-distribution detection. Advances in neural information processing systems. Available online at: https://proceedings.neurips.cc/paper_files/paper/2021/hash/3941c4358616274ac2436eacf67fae05-Abstract.html (accessed June 9, 2025).

[ref27] Fred Agarap A. M. (2018). Deep learning using rectified linear units (ReLU). Available online at: https://arxiv.org/abs/1803.08375v2.

[ref28] Gholamalinezhad H. Khosravi H. (2020). Pooling methods in deep neural networks, a review. Available online at: https://arxiv.org/abs/2009.07485v1.

[ref29] Goodfellow I. Bengio Y. Courville A. (2016). Deep learning. Cambridge, MA, USA: The MIT Press. doi: 10.4258/hir.2016.22.4.351

[ref30] Graves A. (2012) Long short-term memory. Supervised sequence labelling with recurrent neural networks, 37–45. Available online at: 10.1007/978-3-642-24797-2_4.

[ref31] Greaney A. J. Starr T. N. Barnes C. O. Weisblum Y. Schmidt F. Caskey M.. (2021). Mapping mutations to the SARS-CoV-2 RBD that escape binding by different classes of antibodies. Nat. Commun. 12, 4196–4114. doi: 10.1038/s41467-021-24435-8, PMID: 34234131 PMC8263750

[ref32] Gulli A. Pal S. (2017). Deep learning with Keras. Available online at: https://books.google.com/books?hl=en&lr=&id=20EwDwAAQBAJ&oi=fnd&pg=PP1&dq=keras+deep+learning&ots=lJcE9jgOV9&sig=VTXDeuKzKCYykzfji5oijJA2_kM.

[ref33] Gunasekaran H. Ramalakshmi K. Rex Macedo Arokiaraj A. Kanmani S. D. Venkatesan C. Dhas C. S. G. (2021). Analysis of DNA sequence classification using CNN and hybrid models. Comput. Math. Methods Med. 2021:1835056. doi: 10.1155/2021/1835056, PMID: 34306171 PMC8285202

[ref34] Guo C. Pleiss G. Sun Y. Weinberger K. Q. (2017) On calibration of modern neural networks. In: International Conference on Machine Learning. Available online at: http://proceedings.mlr.press/v70/guo17a.html (accessed June 6, 2025).

[ref35] Gupta P. Bagchi A. (2024). Introduction to pandas. In: Essentials of python for artificial intelligence and machine learning Springer Cham, 161–196. Available online at: 10.1007/978-3-031-43725-0_5.

[ref36] Hadfield J. Megill C. Bell S. M. Huddleston J. Potter B. Callender C.. (2018) Nextstrain: real-time tracking of pathogen evolution. Bioinformatics. doi: 10.1093/bioinformatics/bty407 PMC6247931 29790939

[ref37] Hanson G. Adams J. Kepgang D. I. B. Zondagh L. S. Bueh L. T. Asante A.. (2024). Machine learning and molecular docking prediction of potential inhibitors against dengue virus. Front. Chem. 12:1510029. doi: 10.3389/fchem.2024.1510029, PMID: 39776767 PMC11703810

[ref38] Harvey W. T. Carabelli A. M. Jackson B. Gupta R. K. Thomson E. C. Harrison E. M.. (2021). SARS-CoV-2 variants, spike mutations and immune escape. Nat. Rev. Microbiol. 19, 409–424. doi: 10.1038/s41579-021-00573-0, PMID: 34075212 PMC8167834

[ref39] Hochreiter S. Schmidhuber J. (1997). Long short-term memory. Neural Comput. 9, 1735–1780. doi: 10.1162/NECO.1997.9.8.1735 9377276

[ref40] Jacob B. Kligys S. Chen B. Zhu M. Tang M. Howard A.. (2018) Quantization and training of neural networks for efficient integer-arithmetic-only inference. In Proceedings of the IEEE conference on computer vision and pattern recognition. Available online at: http://openaccess.thecvf.com/content_cvpr_2018/html/Jacob_Quantization_and_Training_CVPR_2018_paper.html (accessed July 3, 2025).

[ref41] Juan J. Armenteros A. Kaae Sønderby C. Kaae Sønderby S. Nielsen H. Winther O. (2017). Deeploc: prediction of protein subcellular localization using deep learning. Bioinformatics 33, 3387–3395. doi: 10.1093/bioinformatics/btx431 29036616

[ref42] Karim S. S. A. Karim Q. A. (2021). Omicron SARS-CoV-2 variant: a new chapter in the COVID-19 pandemic. Lancet 398, 2126–2128. doi: 10.1016/S0140-6736(21)02758-6, PMID: 34871545 PMC8640673

[ref43] Katoh K. Rozewicki J. Yamada K. D. (2019). MAFFT online service: multiple sequence alignment, interactive sequence choice and visualization. Brief. Bioinform. 20, 1160–1166. doi: 10.1093/BIB/BBX108, PMID: 28968734 PMC6781576

[ref44] Kingma D. P. Ba J. (2014). Adam: A method for stochastic optimization. In Proceedings of the 3rd International Conference on Learning Representations (ICLR 2015). San Diego, CA, USA: International Conference on Learning Representations (ICLR).

[ref45] Korber B. Fischer W. M. Gnanakaran S. Yoon H. Theiler J. Abfalterer W.. (2020). Tracking changes in SARS-CoV-2 spike: evidence that D614G increases infectivity of the COVID-19 virus. Cell 182, 812–827.e19. doi: 10.1016/j.cell.2020.06.043 32697968 PMC7332439

[ref46] Kramer O., (2016) Scikit-learn. In Machine learning for evolution strategies. Cham: Springer, 20, 45–53. Available online at: 10.1007/978-3-319-33383-0_5.

[ref47] Kull M. Perello-Nieto M. Kängsepp M. Filho T. S. Song H. Flach P. (2019) Beyond temperature scaling: Obtaining well-calibrated multi-class probabilities with dirichlet calibration. Advances in neural information processing systems. Available online at: https://proceedings.neurips.cc/paper/2019/hash/8ca01ea920679a0fe3728441494041b9-Abstract.html (accessed June 6, 2025).

[ref48] Kuzmin K. Adeniyi A. E. DaSouza A. K. Lim D. Nguyen H. Molina N. R.. (2020). Machine learning methods accurately predict host specificity of coronaviruses based on spike sequences alone. Biochem. Biophys. Res. Commun. 533, 553–558. doi: 10.1016/j.bbrc.2020.09.010, PMID: 32981683 PMC7500881

[ref49] Lebatteux D. Soudeyns H. Boucoiran I. Gantt S. Diallo A. B. (2024). Machine learning-based approach KEVOLVE efficiently identifies SARS-CoV-2 variant-specific genomic signatures. PLoS One 19:e0296627. doi: 10.1371/JOURNAL.PONE.0296627, PMID: 38241279 PMC10798494

[ref50] Lecun Y. Bengio Y. Hinton G. (2015). Deep learning. Nature 521, 436–444. doi: 10.1038/NATURE14539, PMID: 26017442

[ref51] Lemenkova P. (2020) Python libraries matplotlib, seaborn and pandas for visualization geospatial datasets generated by QGIS. In: Analele stiintifice ale Universitatii" Alexandru Ioan Cuza" din Iasi-seria Geografie. Available online at: https://hal.science/hal-02949694/ (accessed June 10, 2025).

[ref52] Li N. Cai Q. Miao Q. Song Z. Fang Y. Hu B. (2021). High-throughput metagenomics for identification of pathogens in the clinical settings. Small Methods 5:2000792. doi: 10.1002/SMTD.202000792, PMID: 33614906 PMC7883231

[ref53] Li J. Wu Y. N. Zhang S. Kang X. P. Jiang T. (2022). Deep learning based on biologically interpretable genome representation predicts two types of human adaptation of SARS-CoV-2 variants. Brief. Bioinform. 23:bbac036. doi: 10.1093/bib/bbac036, PMID: 35233612 PMC9116219

[ref54] Liang Q. Bible P. W. Liu Y. Zou B. Wei L. (2020). DeepMicrobes: taxonomic classification for metagenomics with deep learning. NAR Genom. Bioinform. 2:lqaa009. doi: 10.1093/NARGAB/LQAA009, PMID: 33575556 PMC7671387

[ref55] McCarthy K. R. Rennick L. J. Nambulli S. Robinson-McCarthy L. R. Bain W. G. Haidar G.. (2021). Recurrent deletions in the SARS-CoV-2 spike glycoprotein drive antibody escape. Science 371, 1139–1142. doi: 10.1126/science.abf6950, PMID: 33536258 PMC7971772

[ref56] Meng B. Abdullahi A. Ferreira I. A. T. M. Goonawardane N. Saito A. Kimura I.. (2022). Altered TMPRSS2 usage by SARS-CoV-2 omicron impacts infectivity and fusogenicity. Nature 603, 706–714. doi: 10.1038/s41586-022-04474-x, PMID: 35104837 PMC8942856

[ref57] Menzel P. Ng K. L. Krogh A. (2016). Fast and sensitive taxonomic classification for metagenomics with kaiju. Nat. Commun. 7:11257. doi: 10.1038/NCOMMS11257, PMID: 27071849 PMC4833860

[ref58] Nan B. G. Zhang S. Li Y. C. Kang X. P. Chen Y. H. Li L.. (2022). Convolutional neural networks based on sequential spike predict the high human adaptation of SARS-CoV-2 omicron variants. Viruses 14:1072. doi: 10.3390/v14051072, PMID: 35632811 PMC9147419

[ref59] Neyshabur B. Bhojanapalli S. McAllester D. Srebro N. (2017). “Exploring Generalization in Deep Learning” in Proceedings of the 31st Annual Conference on Neural Information Processing Systems (NeurIPS 2017). Long Beach, California, USA: Neural Information Processing Systems Foundation, Inc.

[ref60] Nicora G. Salemi M. Marini S. Bellazzi R. (2022). Predicting emerging SARS-CoV-2 variants of concern through a one class dynamic anomaly detection algorithm. BMJ Health Care Inform. 29:e100643. doi: 10.1136/bmjhci-2022-100643, PMID: 36593658 PMC9742845

[ref61] Nyamari M. N. Omar K. M. Fayehun A. F. Dachi O. Bwana B. K. Awe O. I. (2023). Expression level analysis of ACE2 receptor gene in African-American and non-African-American COVID-19 patients. bioRxiv. doi: 10.1101/2023.09.11.557129

[ref62] Nzungize L. Kengne-Ouafo J. A. Wesonga M. R. Umuhoza D. Murithi K. Kimani P.. (2022). Transcriptional profiles analysis of COVID-19 and malaria patients reveals potential biomarkers in children. bioRxiv. doi: 10.1101/2022.06.30.498338

[ref63] Obura H. O. Mlay C. D. Moyo L. Karumbo B. M. Omar K. M. Sinza E. M.. (2022). Molecular phylogenetics of HIV-1 subtypes in African populations: a case study of sub-Saharan African countries. bioRxiv. doi: 10.1101/2022.05.18.492401

[ref64] Oluwagbemi O. Awe O. I. (2018). A comparative computational genomics of Ebola virus disease strains: in-silico insight for Ebola control. Inform. Med. Unlocked 12, 106–119. doi: 10.1016/j.imu.2018.07.004

[ref65] Omar K. M. Kitundu G. L. Jimoh A. O. Namikelwa D. N. Lisso F. M. Babajide A. A.. (2024). Investigating antimicrobial resistance genes in Kenya, Uganda and Tanzania cattle using metagenomics. PeerJ 12:e17181. doi: 10.7717/PEERJ.17181/SUPP-9 38666081 PMC11044882

[ref66] Pawar S. V. Banini W. S. K. Shamsuddeen M. M. Jumah T. Dolling N. N. Tiamiyu A.. (2024). Prostruc: an open-source tool for 3D structure prediction using homology modeling. Front. Chem. 12:1509407. doi: 10.3389/fchem.2024.1509407, PMID: 39717221 PMC11664737

[ref67] Qu J. Chen Y. Yue X. Fu W. Huang Q. (2024). Hyper-opinion evidential deep learning for out-of-distribution detection. Advances in Neural Information Processing Systems. Available online at: https://proceedings.neurips.cc/paper_files/paper/2024/hash/99d4ceebdf75b64e8ed608a245b63416-Abstract-Conference.html (accessed June 9, 2025).

[ref68] Ramasubramanian K. Singh A. (2019) Deep learning using keras and tensorflow. Machine Learning Using R: With Time Series and Industry-Based Use Cases in R Springer, 667–688. Available online at: 10.1007/978-1-4842-4215-5_11.

[ref69] Randhawa G. S. Soltysiak M. P. M. El Roz H. de Souza C. P. E. Hill K. A. Kari L. (2020). Machine learning using intrinsic genomic signatures for rapid classification of novel pathogens: COVID-19 case study. PLoS One 15:e0232391. doi: 10.1371/JOURNAL.PONE.0232391, PMID: 32330208 PMC7182198

[ref70] Reimers N., &Gurevych I. (2017). Reporting score distributions makes a difference: performance study of LSTM-networks for sequence tagging. EMNLP 2017 – Conference on Empirical Methods in Natural Language Processing, Proceedings, 338–348. Available online at: 10.18653/v1/d17-1035.

[ref71] Ren J. Ahlgren N. A. Lu Y. Y. Fuhrman J. A. Sun F. (2017). Virfinder: a novel k-mer based tool for identifying viral sequences from assembled metagenomic data. Microbiome 5:69. doi: 10.1186/s40168-017-0283-5 28683828 PMC5501583

[ref72] Roux S. Enault F. Hurwitz B. L. Sullivan M. B. (2015). VirSorter: mining viral signal from microbial genomic data. PeerJ 2015:e985. doi: 10.7717/PEERJ.985/SUPP-2 PMC4451026 26038737

[ref73] Saito A. Irie T. Suzuki R. Maemura T. Nasser H. Uriu K.. (2022). Enhanced fusogenicity and pathogenicity of SARS-CoV-2 delta P681R mutation. Nature 602, 300–306. doi: 10.1038/S41586-021-04266-9, PMID: 34823256 PMC8828475

[ref74] Samek W. Montavon G. Lapuschkin S. Anders C. J. Müller K. R. (2021). Explaining deep neural networks and beyond: A review of methods and applications. Proceedings of the IEEE. Available online at: 10.1109/JPROC.2021.3059260.

[ref75] Sergeev A. Del Balso M. (2018). Horovod: fast and easy distributed deep learning in TensorFlow. Available online at: https://arxiv.org/pdf/1802.05799.

[ref76] Srivastava N. Hinton G. Krizhevsky A. Salakhutdinov R. (2014). Dropout: a simple way to prevent neural networks from overfitting. J. Mach. Learn. Res. 15, 1929–1958. Available online at: https://dl.acm.org/doi/abs/10.5555/2627435.2670313

[ref77] Sukhorukov G. Khalili M. Gascuel O. Candresse T. Marais-Colombel A. Nikolski M. (2022). VirHunter: a deep learning-based method for detection of novel RNA viruses in plant sequencing data. Front. Bioinform. 2:867111. doi: 10.3389/fbinf.2022.867111, PMID: 36304258 PMC9580956

[ref78] Tampuu A. Bzhalava Z. Dillner J. Vicente R. (2019). ViraMiner: deep learning on raw DNA sequences for identifying viral genomes in human samples. PLoS One 14:e0222271. doi: 10.1371/JOURNAL.PONE.0222271, PMID: 31509583 PMC6738585

[ref79] Vaicenavicius J. Widmann D. Andersson C. Lindsten F. Roll J. Schön T. (2019). Evaluating model calibration in classification. The 22nd international conference on artificial intelligence and statistics. Available online at: https://proceedings.mlr.press/v89/vaicenavicius19a (accessed June 6, 2025).

[ref80] Wang M. Lu S. Zhu D. Lin J. Wang Z. (2018). A high-speed and low-complexity architecture for softmax function in deep learning. 2018 IEEE Asia Pacific Conference on Circuits and Systems (APCCAS). Available online at: 10.1109/APCCAS.2018.8605654.

[ref81] Whata A. Chimedza C. (2021). Deep learning for SARS COV-2 genome sequences. IEEE Access 9, 59597–59611. doi: 10.1109/ACCESS.2021.3073728, PMID: 34812391 PMC8545213

[ref82] Zeng H. Edwards M. D. Liu G. Gifford D. K. (2016). Convolutional neural network architectures for predicting DNA–protein binding. Bioinformatics 32, i121–i127. doi: 10.1093/BIOINFORMATICS/BTW255 27307608 PMC4908339

[ref83] Zhang S. Zheng D. Hu X. Yang M.. (2015) Bidirectional long short-term memory networks for relation classification. Proceedings of the 29th Pacific Asia conference on language, information and computation. Available online at: https://aclanthology.org/Y15-1009.pdf.

[ref84] Zhou P. Yang X. Lou W. Wang X. G. Hu B. Zhang L.. (2020). A pneumonia outbreak associated with a new coronavirus of probable bat origin. Nature 579, 270–273. doi: 10.1038/S41586-020-2012-7 32015507 PMC7095418

