# Supplementary material for: Enhanced deep Convolutional Neural Network for SARS-CoV-2 variants classification
Source: Front Artif Intell. 2025 Sep 8;8:1512003. doi: 10.3389/frai.2025.1512003 (PMC12450893; doi:10.3389/frai.2025.1512003)
Supplement: Supplementary file 1 [file Data_Sheet_1.docx]

**Enhanced Deep Convolutional Neural Network for SARS-CoV-2 Variants Classification**

Olaitan I. Awe^1,2^, Hesborn O. Obura^3,4^, Charles Ssemuyiga^5,6*^, Evans Mudibo^3,4,7^ and Mike J. Mwanga^3,4^

^1^African Society for Bioinformatics and Computational Biology, Cape Town, South Africa

^2^Department of Computer Science, Faculty of Science, University of Ibadan, Oyo State, Nigeria

^3^Department of Biochemistry and Biotechnology, School of Pure and Applied Science, Pwani University, Kilifi, Kenya

^4^Pwani University Biosciences Research Centre, Pwani University, Kilifi, Kenya

^5^PharmaQsar Bioinformatics Firm, Kampala, Uganda.

^6^Department of Biological and Environmental Sciences, School of Natural Sciences, Kampala International University, Kampala, Uganda

^7^Centre for Geographic Medicine Research, Kenya Medical Research Institute Wellcome Trust Research Program, Kilifi, Kenya

Table S1: Distribution of SARS-CoV-2 variants in the training and validation datasets used for model development. Counts represent the number of genome sequences per variant.

| Variant | Training Set | Validation Set | Total |
| --- | --- | --- | --- |
| Alpha | 5,511 | 2,317 | 7,828 |
| Beta | 4,271 | 2,016 | 6,287 |
| Gamma | 5,597 | 1,981 | 7,578 |
| Delta | 5,495 | 1,688 | 7,183 |
| Omicron | 6,362 | 583 | 6,945 |
| Total | 27,236 | 8,585 | 35,821 |


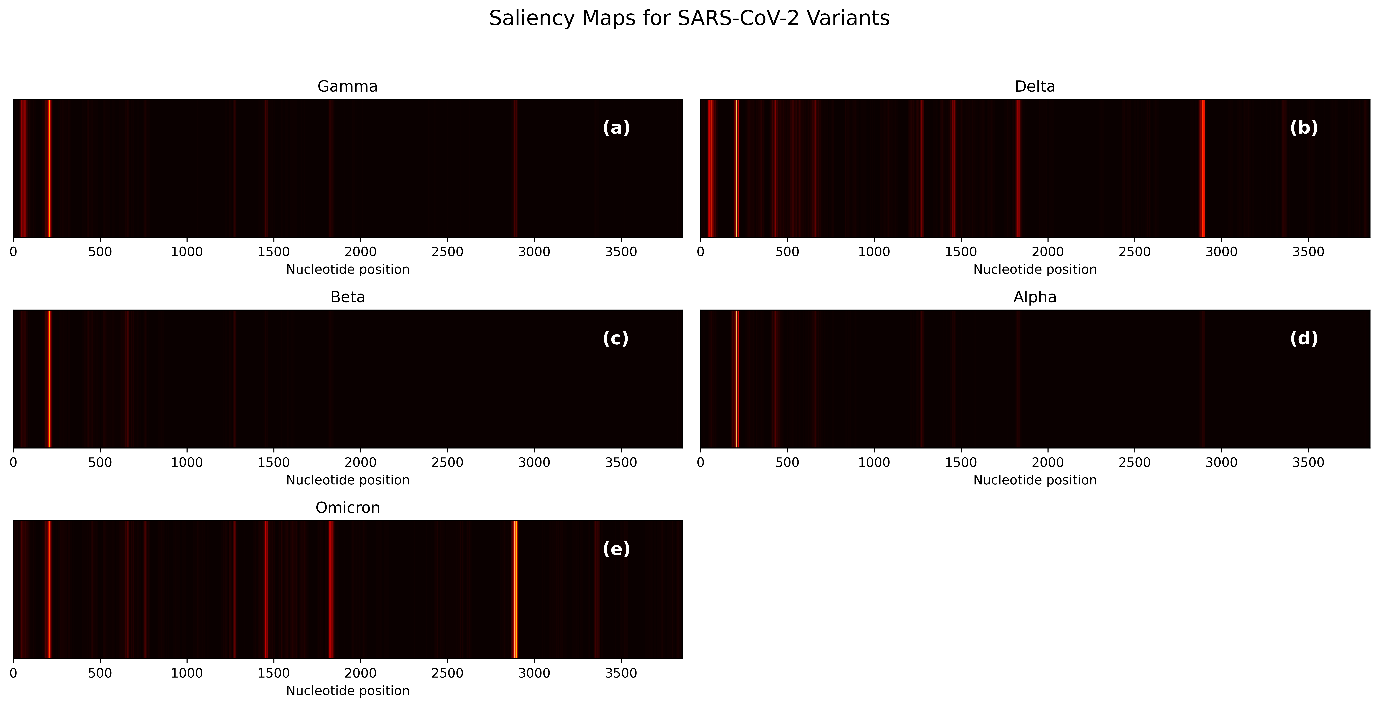


Figure S1: Saliency Maps at a single nucleotide stamp. Regions with higher saliency indicate positions most influential in classification.


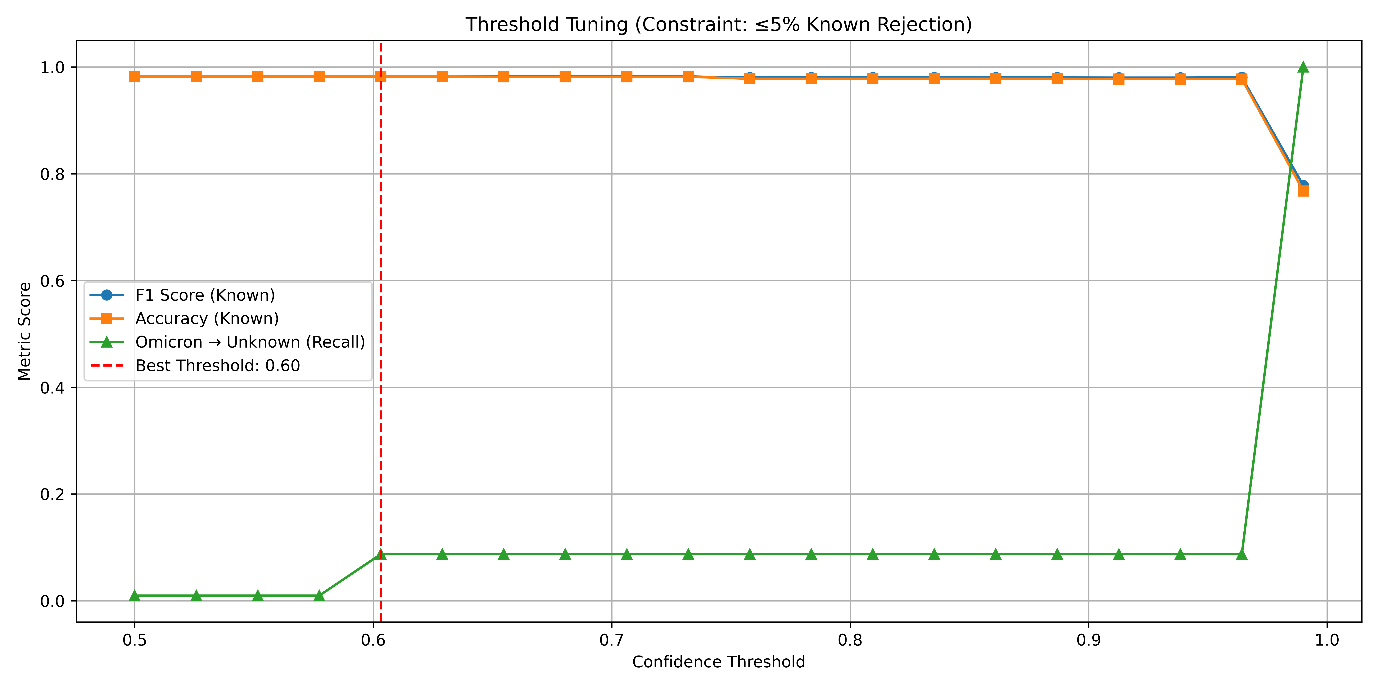


Figure S2: Threshold tuning analysis for unknown variant rejection. A maximum constraint of 5% rejection of known classes was applied, ensuring model robustness in detecting novel or unseen variants.


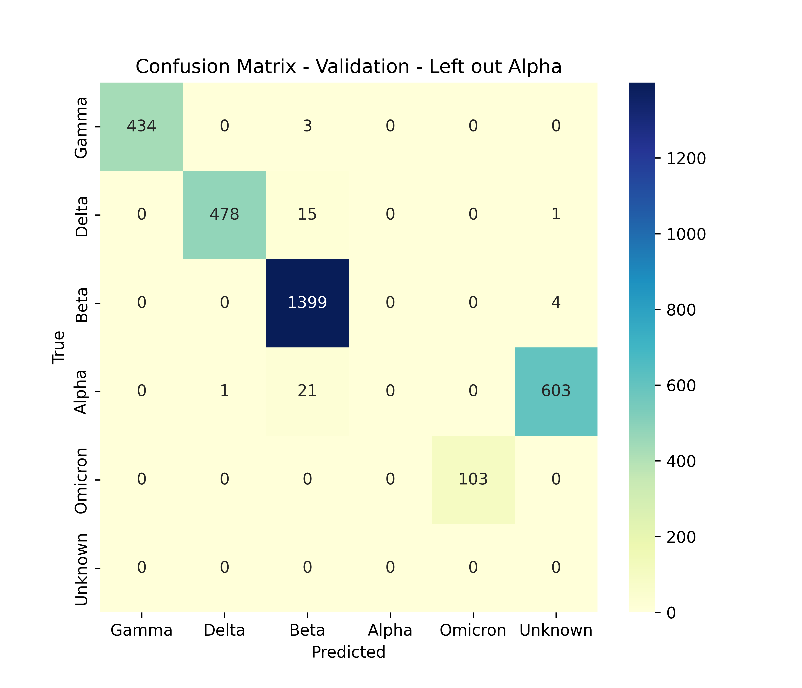


Figure S3: Confusion matrix for LOVO experiment excluding Alpha variant.
All other variants, including Gamma and Delta, maintain strong class separation.


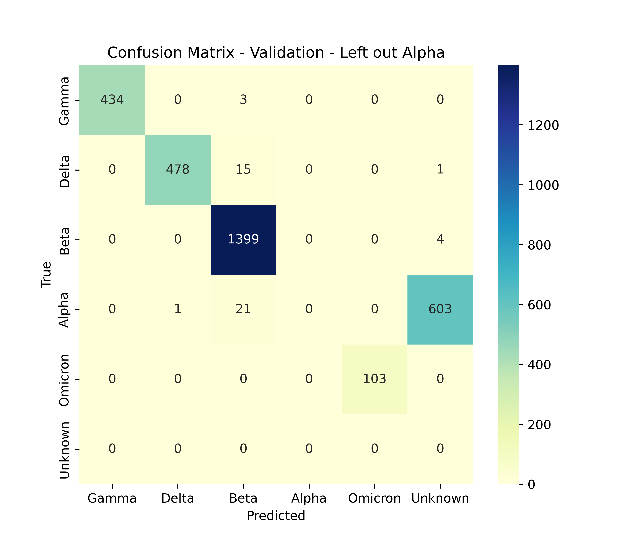
Table S2: Classification metrics for the LOVO experiment excluding Alpha during training. Both macro and weighted averages are reduced, confirming limited generalization when Alpha is absent.

| Variant | Precision | Recall | F1-Score | Support |
| --- | --- | --- | --- | --- |
| Gamma | 1.00 | 0.99 | 1.00 | 437 |
| Delta | 0.98 | 0.97 | 0.97 | 494 |
| Beta | 0.69 | 1.00 | 0.82 | 1403 |
| Alpha | 0.00 | 0.00 | 0.00 | 625 |
| Omicron | 1.00 | 1.00 | 1.00 | 103 |
|  |  |  |  |  |
| Accuracy |  |  | 0.79 | 3062 |
| Macro avg | 0.73 | 0.79 | 0.76 | 3062 |
| Weighted avg | 0.65 | 0.79 | 0.71 | 3062 |


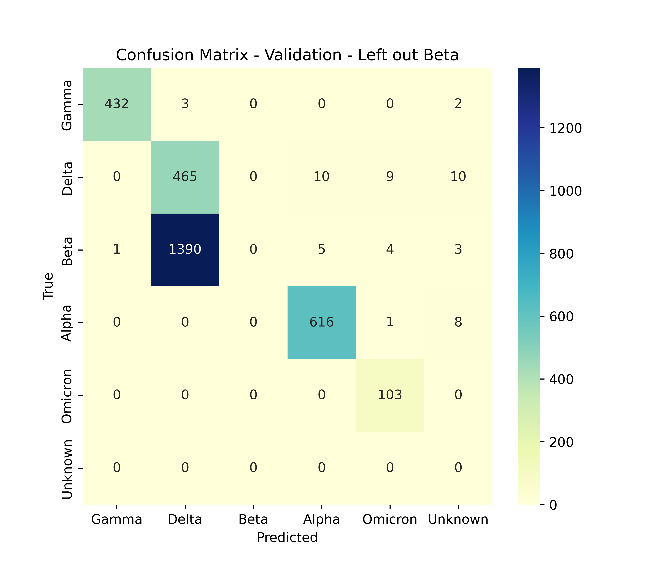


Figure S4: Confusion matrix for LOVO experiment excluding Beta variant.
Strong diagonal signals remain for other classes.

| Variant | Precision | Recall | F1-Score | Support |
| --- | --- | --- | --- | --- |
| Gamma | 1.00 | 0.99 | 0.99 | 437 |
| Delta | 0.25 | 0.94 | 0.40 | 494 |
| Beta | 0.00 | 0.00 | 0.00 | 1403 |
| Alpha | 0.98 | 0.99 | 0.98 | 625 |
| Omicron | 0.74 | 1.00 | 0.85 | 103 |
|  |  |  |  |  |
| Accuracy |  |  | 0.53 | 3062 |
| Macro avg | 0.59 | 0.78 | 0.64 | 3062 |
| Weighted avg | 0.41 | 0.53 | 0.43 | 3062 |

Table S3: Classification report for LOVO experiment with Beta excluded during training. The macro average indicates moderate class imbalance and highlights limited adaptability to unseen variants.


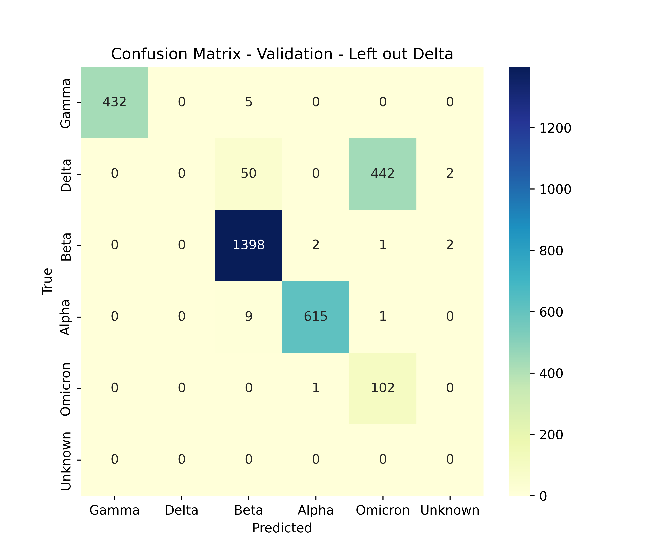


Figure S5: Confusion matrix for LOVO experiment excluding Delta variant.

| Variant | Precision | Recall | F1-Score | Support |
| --- | --- | --- | --- | --- |
| Gamma | 1.00 | 0.99 | 0.99 | 437 |
| Delta | 0.00 | 0.00 | 0.00 | 494 |
| Beta | 0.96 | 1.00 | 0.98 | 1403 |
| Alpha | 1.00 | 0.98 | 0.99 | 625 |
| Omicron | 0.19 | 0.99 | 0.31 | 103 |
|  |  |  |  |  |
| Accuracy |  |  | 0.83 | 3062 |
| Macro avg | 0.63 | 0.79 | 0.65 | 3062 |
| Weighted avg | 0.79 | 0.83 | 0.80 | 3062 |

Table S4: Classification metrics for LOVO experiment excluding Delta during training. Performance is impaired for Delta and Omicron, though other variants retain high accuracy.


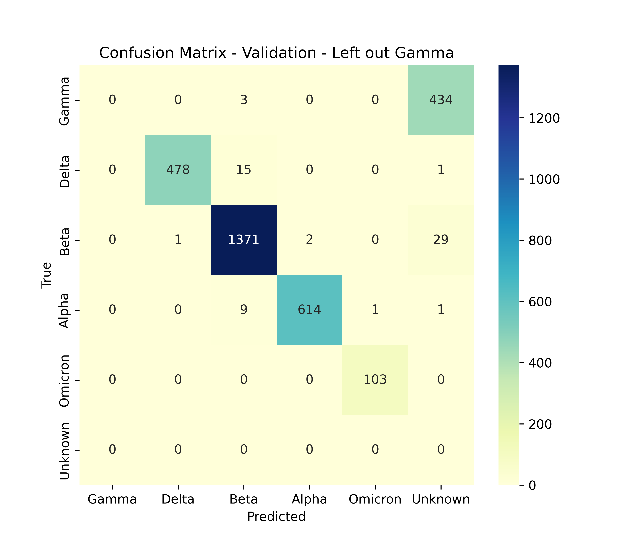


Figure S6: Confusion matrix for LOVO experiment excluding Gamma variant.

Table S5: Classification metrics for LOVO experiment with Gamma excluded during training. Poor generalization to Gamma is evident, while other variants remain well classified.

| Variant | Precision | Recall | F1-Score | Support |
| --- | --- | --- | --- | --- |
| Gamma | 0.00 | 0.00 | 0.00 | 437 |
| Delta | 1.00 | 0.97 | 0.98 | 494 |
| Beta | 0.98 | 0.98 | 0.98 | 1403 |
| Alpha | 1.00 | 0.98 | 0.99 | 625 |
| Omicron | 0.99 | 1.00 | 1.00 | 103 |
| Unknown | 0.00 | 0.00 | 0.00 | 0 |
|  |  |  |  |  |
| Accuracy |  |  | 0.84 | 3062 |
| Macro avg | 0.66 | 0.65 | 0.66 | 3062 |
| Weighted avg | 0.85 | 0.84 | 0.84 | 3062 |


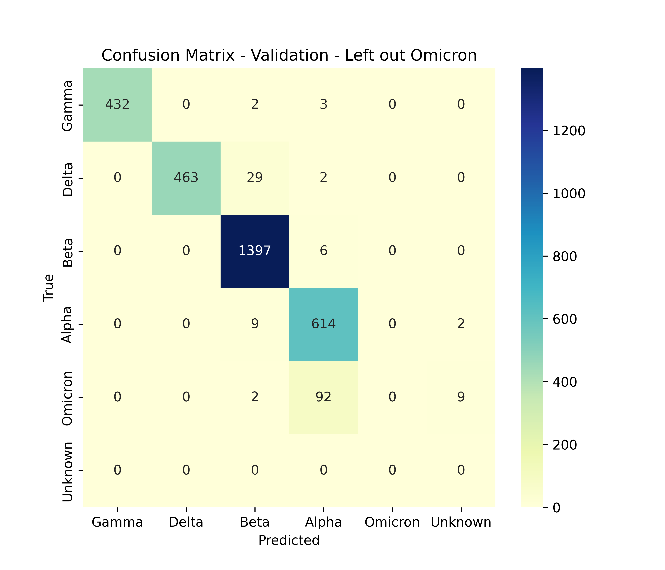


Figure S7: Confusion matrix for LOVO experiment excluding Omicron variant.

Table S6: Classification metrics for LOVO experiment excluding Omicron during training. Model generalization fails for Omicron but remains high for other variants.

| Variant | Precision | Recall | F1-Score | Support |
| --- | --- | --- | --- | --- |
| Gamma | 1.00 | 0.99 | 0.99 | 437 |
| Delta | 1.00 | 0.94 | 0.97 | 494 |
| Beta | 0.97 | 1.00 | 0.98 | 1403 |
| Alpha | 0.86 | 0.98 | 0.92 | 625 |
| Omicron | 0.00 | 0.00 | 0.00 | 103 |
| Unknown | 0.00 | 0.00 | 0.00 | 0 |
|  |  |  |  |  |
| Accuracy |  |  | 0.95 | 3062 |
| Macro avg | 0.64 | 0.65 | 0.64 | 3062 |
| Weighted avg | 0.92 | 0.95 | 0.94 | 3062 |

Table S7: Translation details of key SARS-CoV-2 spike protein mutations used in the feature importance analysis. Includes amino acid changes, codon substitutions, nucleotide positions, and corresponding DNA sequences.

| Mutation | Amino Acid Change | Reference Codon(s) | Mutated Codon(s) | Nucleotide Position (Approx.) | Corresponding DNA Sequence (5' → 3') |
| --- | --- | --- | --- | --- | --- |
| N501Y | N (Asparagine) → Y (Tyrosine) | AAT | TAT | 23,063 - 23,065 | AAT → TAT |
| E484K | E (Glutamic acid) → K (Lysine) | GAA, GAG | AAA, AAG | 23,024 - 23,026 | GAA/GAG → AAA/AAG |
| E484Q | E (Glutamic acid) → Q (Glutamine) | GAA, GAG | CAA, CAG | 23,024 - 23,026 | GAA/GAG → CAA/CAG |
| K417N | K (Lysine) → N (Asparagine) | AAA | AAT | 22,841 - 22,843 | AAA → AAT |
| K417T | K (Lysine) → T (Threonine) | AAA | ACT | 22,841 - 22,843 | AAA → ACT |


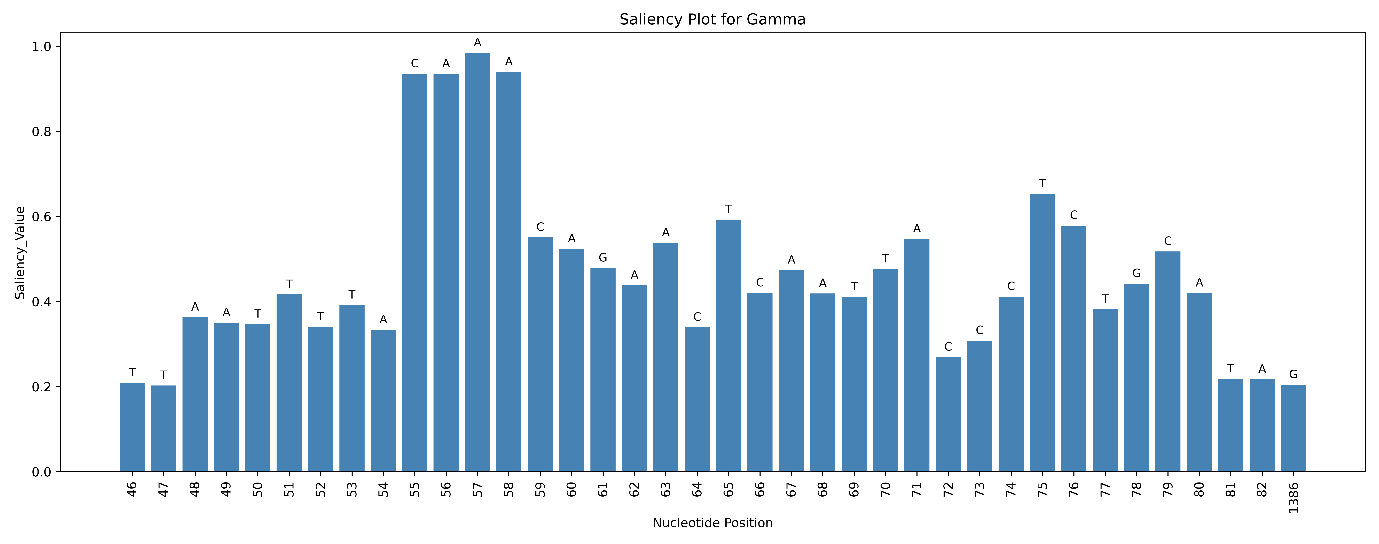


Figure S8: Variant-specific saliency plot for Gamma sequences, highlighting nucleotide positions most critical for classification.
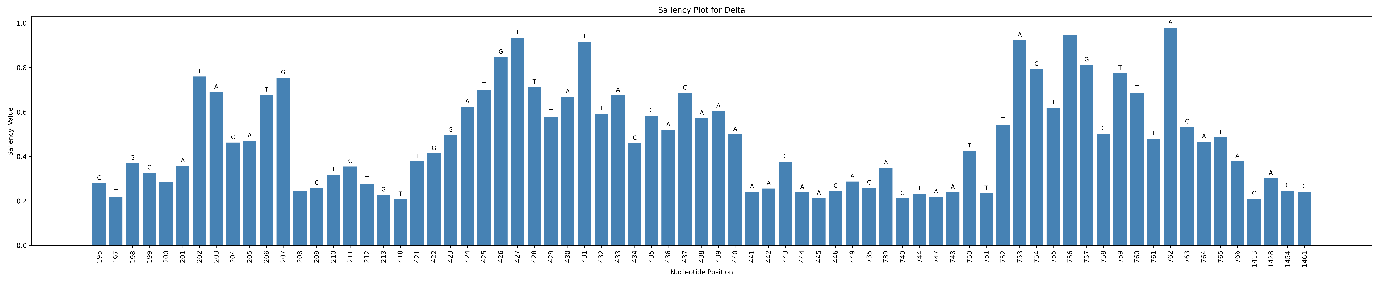


Figure S9: Variant-specific saliency plot for Delta sequences, highlighting nucleotide positions most critical for classification.


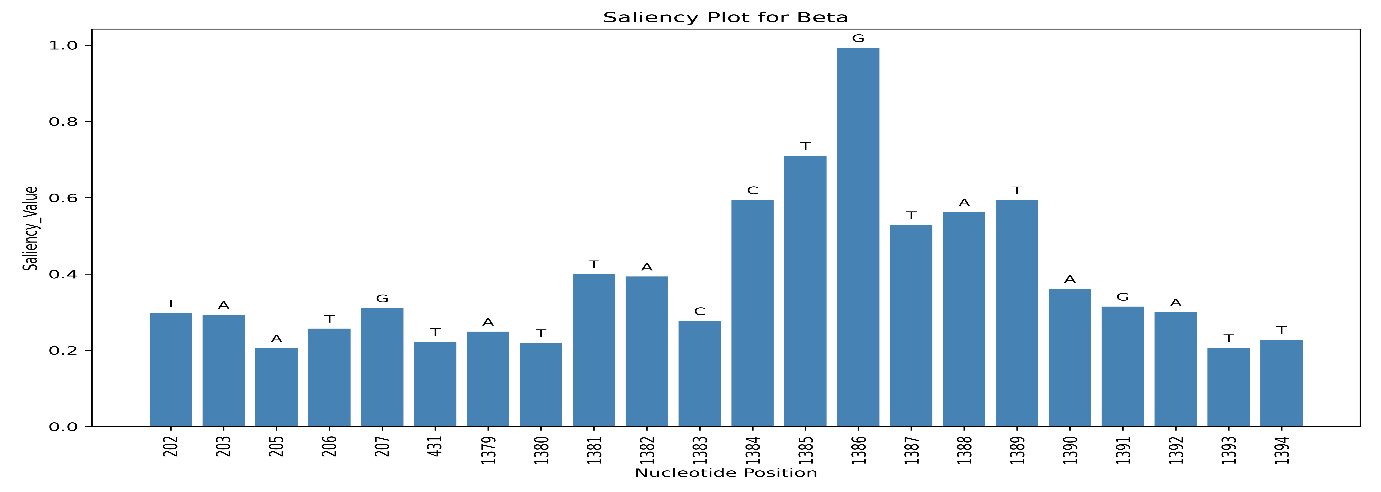


Figure S10: Variant-specific saliency plot for Beta sequences, highlighting nucleotide positions most critical for classification.


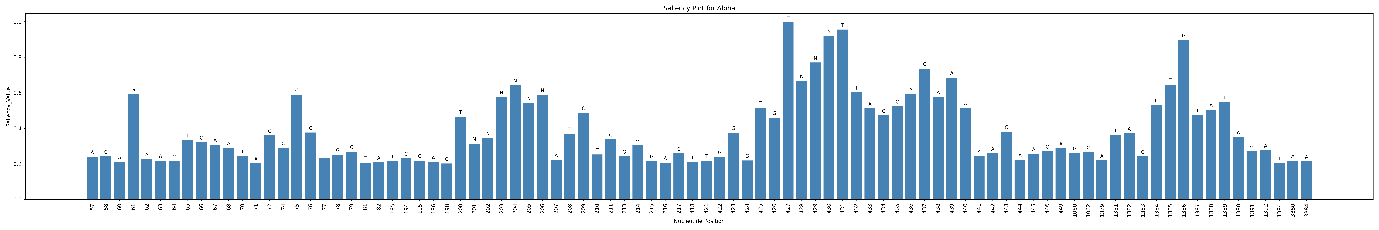


Figure S11: Variant-specific saliency plot for Alpha sequences, highlighting nucleotide positions most critical for classification.


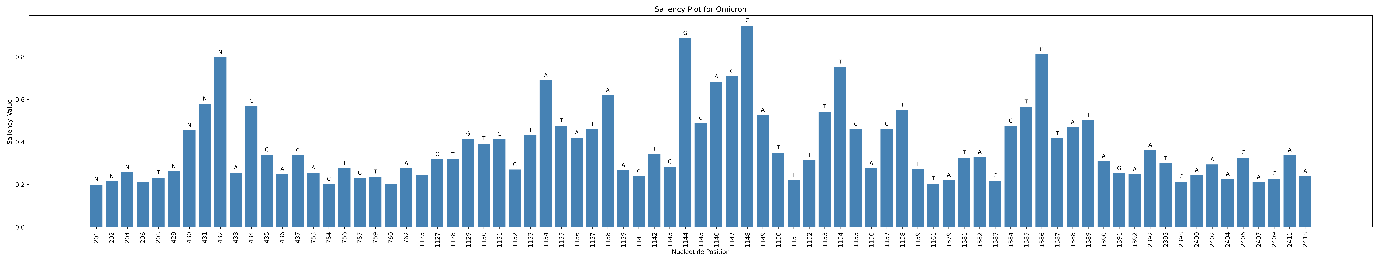


Figure S12: Variant-specific saliency plot for Omicron sequences, highlighting nucleotide positions most critical for classification.
